# MicroRNA Predictors of Longevity in *Caenorhabditis elegans*

**DOI:** 10.1371/journal.pgen.1002306

**Published:** 2011-09-29

**Authors:** Zachary Pincus, Thalyana Smith-Vikos, Frank J. Slack

**Affiliations:** Department of Molecular, Cellular, and Developmental Biology, Yale University, New Haven, Connecticut, United States of America; Stanford University Medical Center, United States of America

## Abstract

Neither genetic nor environmental factors fully account for variability in individual longevity: genetically identical invertebrates in homogenous environments often experience no less variability in lifespan than outbred human populations. Such variability is often assumed to result from stochasticity in damage accumulation over time; however, the identification of early-life gene expression states that predict future longevity would suggest that lifespan is least in part epigenetically determined. Such “biomarkers of aging,” genetic or otherwise, nevertheless remain rare. In this work, we sought early-life differences in organismal robustness in unperturbed individuals and examined the utility of microRNAs, known regulators of lifespan, development, and robustness, as aging biomarkers. We quantitatively examined *Caenorhabditis elegans* reared individually in a novel apparatus and observed throughout their lives. Early-to-mid–adulthood measures of homeostatic ability jointly predict 62% of longevity variability. Though correlated, markers of growth/muscle maintenance and of metabolic by-products (“age pigments”) report independently on lifespan, suggesting that graceful aging is not a single process. We further identified three microRNAs in which early-adulthood expression patterns individually predict up to 47% of lifespan differences. Though expression of each increases throughout this time, *mir-71* and *mir-246* correlate with lifespan, while *mir-239* anti-correlates. Two of these three microRNA “biomarkers of aging” act upstream in insulin/IGF-1–like signaling (IIS) and other known longevity pathways, thus we infer that these microRNAs not only report on but also likely determine longevity. Thus, fluctuations in early-life IIS, due to variation in these microRNAs and from other causes, may determine individual lifespan.

## Author Summary

Why do some individuals live longer than others? Unexpectedly, genetic differences contribute surprisingly little to lifespan variation in humans. The situation is thrown into relief in studies of *C. elegans*, in which genetically identical siblings reared in identical environments usually experience different lifespans. In this work, we show that physiological differences between identical animals begin to appear relatively early in life and that markers of ill health in young adulthood presage shorter lifespans. Using fluorescent markers to examine the level of activation of several genes, we found three regulatory microRNA genes that vary in activity between individuals in a manner that predicts future lifespan. Moreover, two of these regulate insulin signaling, which is well known to alter lifespan in diverse species when experimentally manipulated. This indicates that different levels of insulin signaling in otherwise identical individuals may determine differences in lifespan.

## Introduction

Inter-individual variation in human longevity has not been found to be under substantial genetic control, with heritability generally between 15% and 30% [1],[2]. At the same time, shared environmental factors contribute little in these human studies, and can be completely controlled in large-scale experiments on inbred invertebrates without abrogating lifespan variability [3]–[5]. Indeed, rearing *Caenorhabditis elegans* in a homogenous, chemically defined liquid medium more than doubles the coefficient of variability in lifespan compared to feeding the animals live bacteria on solid agar (a less-homogenous environment) [6]. As the external environment of *C. elegans* can be easily controlled, the genetics of its lifespan are well understood [7], and its developmental plan is famously invariant, this nematode is an ideal organism in which to investigate how and when individuality arises, and how these differences produce a phenotype as variable as lifespan [8].

The identification of “biomarkers of longevity” – measurable parameters that predict individual longevity better than chronological age [9] – will help pinpoint genetic and physiological processes that promote or defer senescent decline. Further, such biomarkers may help clarify whether lifespan differences are simply the result of variable accumulation of damage over time, or whether they may also result from gene-regulatory states, potentially set early in life, that determine individual robustness [10]. To date, most identified and proposed biomarkers in *C. elegans* have largely been phenomenological, downstream indicators of homeostatic maintenance. One important class of such markers is locomotory function. Herndon and colleagues showed that a qualitative evaluation of individual locomotory ability correlates with remaining lifespan of same-aged animals, and, moreover, that these movement classes correlate with the degree of sarcopenia (decline in muscle mass and function) in those individuals [11]. Later work showed quantitative correlations between the rate of decrease in body movement and lifespan [12], as well as between the span of functional pharyngeal pumping or body movement and lifespan [13], [14]. In addition to muscle decline, general decreases in macromolecular homeostasis have long been observed in aging *C. elegans* via increases in non-hydrolysable, autofluorescent “age pigments” such as lipofuscin [15] in intestinal lysosomes [16]–[18]. Lipofuscin accumulation correlates with the qualitative movement classes defined in Herndon *et al.* [19], though such accumulation has not been directly shown to predict an individual's future longevity, and in one recent work was specifically not found be predictive of longevity [20]. (This last observation was made of green-wavelength autofluorescence, which is more specific for flavin compounds, while lipofuscin *per se* fluoresces most strongly in blue wavelengths [19], [21].) Lastly, animals that reach their final adult size more rapidly have shorter lifespans [14]. As part of this work, we systematically validated adult growth and movement rates, tissue homeostasis, and age pigment accumulation as phenomenological biomarkers of longevity in nematodes, and further, by measuring multiple biomarkers per individual, deduced the relationships among these markers.

While they may suggest clinically relevant markers of human aging, such measurements do little to elucidate the genetic mechanisms underlying lifespan variability. Transcriptional profiling of aging *C. elegans* has suggested sets of genes that change expression during aging and may thus report an animal's “physiological age” [22]–[24]. The definitive test remains demonstrating that a particular gene's expression level predicts future longevity on an individual basis. The first genetic predictor of individual lifespan was identified by Rea and colleagues, who demonstrated that the ability to upregulate a reporter for expression of the heat-shock protein *hsp-16.2* after a mild heat stress correlates with post-stress longevity [25]. However, such stress also induces a protective effect [26]. Thus it is not clear whether the measured effect reflects innate differences in “heat-shock response capacity”, which in un-stressed animals might also correlate with future longevity, or whether the degree of heat-shock response is determined stochastically at the time of the stress. Recently, mid-life expression variation in several additional genes in *C. elegans,* including *daf-16* and its well-known target *sod-3*, have been shown to predict future longevity in un-perturbed individuals [20]. Therefore, we sought regulatory factors further upstream that might have constitutive activities that determine robustness to damage and/or longevity in unperturbed animals.

MicroRNAs (miRNAs) – short non-coding RNAs that bind to and regulate the expression of target mRNAs – have been proposed as determinants of organismal robustness to environmental variation [27], a prediction that has been borne out experimentally [28], [29]. Similarly, miRNAs may regulate longevity by determining individual capacities to respond to damage [30]. *lin-4* was the first miRNA to be shown to regulate lifespan and stress-resistance, through its action on the insulin/IGF-1-like signaling (IIS) pathway [31], which is well known for its role in longevity determination [32]–[34]. Many miRNAs change expression levels during aging in *C. elegans* [35], and recently *mir-71*, *mir-239*, and *mir-246*, all of which increase in expression over time, have been shown to promote (*mir-71*, *mir-246*) and antagonize (*mir-239*) longevity and stress-resistance, through IIS (*mir-71*, *mir-239*) and the DNA damage response pathway (*mir-71*) [36]. Further, miRNAs in other contexts have proven to be able biomarkers of various human pathologies [37]–[40] and perhaps also aging [41]. Here we report that *mir-71*, *mir-239*, and *mir-246* expression profiles, measured by promoter::GFP reporter constructs, predict individual longevity in *C. elegans*.

## Results

### Lifelong Observation of Individual *C. elegans*

**Figure 1:**
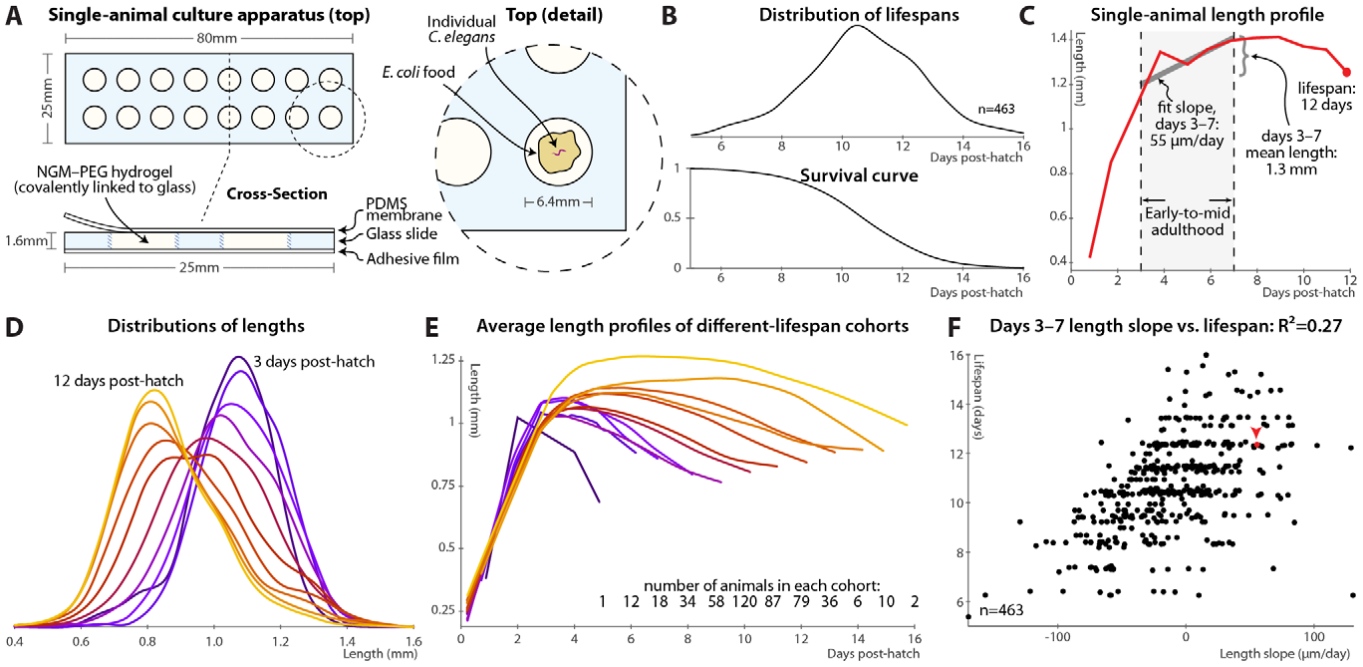
Single-animal vermiculture and measurement. (A) Individual *C. elegans* and their bacterial food source live atop a gel pad, sealed with a gas-permeable polydimethylsiloxane (PDMS) membrane. (B) Variability in individual lifespans is clear from the distribution of lifespans of 463 animals reared in this apparatus, reconstructed via kernel density estimation. The corresponding survival curve is shown below the lifespan distribution, and represents precisely the same data. We prefer the distribution, as features such as bimodality and differences in variance are easier to identify. (C) Time-course of measured length for a single individual throughout its life. Early-to-mid-adulthood patterns in this (and other) measurements are summarized as the average level between days 3 and 7, and the slope of a least-squares fit line to the data in that range. (D) Kernel density estimates of the distribution of lengths of animals at different ages post-hatch, colored by age on a blue-red-yellow spectrum, demonstrate a general shrinkage with aging. (E) The average length over time is shown for cohorts of animals, grouped according to the number of days lived. Shorter-lived animals are in general smaller and shrink in size more quickly. (F) Size maintenance during adulthood (measured as the slope of the least-squares fit of age vs. length, days 3–7 post-hatch) correlates with eventual lifespan; R^2^ = 0.27 (*p*<10^−33^); the leave-one-out (l.o.o.) estimate of future predictive ability is also 0.27. The point corresponding to the individual in panel B is shown in red and marked with an arrowhead. Multivariate regression of lifespan against both length slope and days 3–7 mean length yields an R^2^ of 0.32 (*p*<10^−38^; l.o.o. 0.31).

To determine early-life correlates of eventual longevity, we developed a minimally invasive individual-nematode culture system (Figure 1A) that allows *in situ* imaging of freely moving, unanesthetized animals. Briefly, single eggs at the pre-hatch “pretzel” stage and a bacterial food source are deposited atop PEG-1000-methacrylate hydrogel pads embedded in and crosslinked to a glass slide (see Materials and Methods). The top of the slide is covered with liquid polydimethylsiloxane (PDMS), which polymerizes in approximately 12 hours at 23°C to yield a thin, transparent, and gas-permeable membrane that reduces desiccation and prevents contamination. (All ages reported in this work refer to time after slide preparation; as approximately 98% of viable eggs hatched within 5 hours, we simply report this as “age post-hatch.”) All strains were crossed into the temperature-sensitive fertility-defective strain *spe-9(hc88)* and all assays were conducted at 23°C to prevent reproduction [42]. We obtained good developmental synchrony with this method; after 40 hours, most animals are near the middle of the 4^th^-larval stage, based on vulval morphology (not shown). The mean lifespan of 10.7 days at 23°C in this apparatus is similar to that in standard culture conditions, according to previous reports and our own controls (see Materials and Methods).

At each timepoint, brightfield/fluorescence image pairs were acquired for each animal, and movement rates and health status evaluated by examining motion after stimulation with 0.25 seconds of green light; animals that did not respond were deemed to have perished (Figure S1). Figure 1B illustrates the distribution of lifespans of 463 individuals cultured in this apparatus and the corresponding survival curve.

In this fashion, measurements can be made on individual animals throughout their lives and correlated with eventual longevity. In particular, the position of each animal was identified in brightfield images via custom semi-automated software, allowing quantification of various morphological and image-based features. As an example, Figure 1C illustrates the length of one particular animal measured at daily intervals from hatching until death. In attempting to determine correlates of future longevity, we focused on measurements made during days 3–7 post-hatch, stretching from the attainment of adulthood (beginning of the reproductive period) to the onset of mortality. Less than 3% of the animals die before day 7; while measurements made later often correlate better with remaining longevity, they are of more limited utility as more of the study population has died before the measurements could be made. The day 3–7 range is illustrated in Figure 1C along with the two measures we employed to summarize data in this range: the mean level of a particular measurement over that span, and the slope of a least-squares linear fit of the data in that span.

As noted previously [14], we observed that adult *C. elegans* tend to shrink over time (Figure 1D). This shortening is not observed in the length distributions of heat-killed animals of different ages [43]; we also anecdotally observed that animals often “relax” and lengthen after death. Thus, the shortening appears to reflect a physiological process and not an actual change in the size of the cuticle. As such, we wished, as an illustrative test case, to examine whether size and/or size maintenance over time had any relevance for eventual lifespan. Figure 1E illustrates a retrospective analysis: the average length-versus-time profile is shown for animals grouped according to the number of days lived. Quite clearly, longer-lived animals are both markedly larger than their short-lived siblings and better able to maintain their length over time. This analysis can be made prospectively as well: the slope of the length vs. time curve between days 3 and 7 (as per Figure 1C) correlates well with each animal's future longevity (Figure 1F). Specifically, 27% of overall lifespan variability is accounted for by the days 3–7 length slope alone. (This correlation, and all others shown, is the aggregate of several trials, described in Table S1; per-trial results are given in Figure S3.) The mean length over that time range also correlates positively with lifespan; including both in the regression analysis increases the R^2^ measure of lifespan-predictive ability to 32%. We found similar correlations with volume and surface area; however, length is the most robust.

Note that the R^2^ value is often an over-optimistic estimate of how well a model will predict values from future data, due to “over-fitting” of particular features of the original dataset, particularly with least-squares models, small or outlier-prone datasets, and/or multiple independent parameters. We therefore also estimated future predictive ability via leave-one-out (l.o.o.) cross-validation, in which the prediction for each data point is generated from a model constructed using all other data points. For the length measurements the l.o.o. R^2^ is 31%.

Finally, regression models predict lifespan quantitatively; we can simplify this to a categorical measure to ask how well above- or below-average *predicted* longevity translates to *actual* longevity. Figure S2A shows the distributions of observed lifespans for animals with above-average and below-average predicted longevity based the two length measurements (days 3–7 slope and mean); Figure S2B illustrates the corresponding survival curves. We find that above-average length-predicted lifespan is 71% sensitive and specific for above-average longevity. (Defining the test about the average predicted and measured values yields balanced sensitivity and specificity; other thresholds trade off between the two.) The above-average-predicted-lifespan cohort has a 17% increase in mean lifespan compared to the below-average cohort.

### Phenomenological Predictors of Longevity

**Figure 2:**
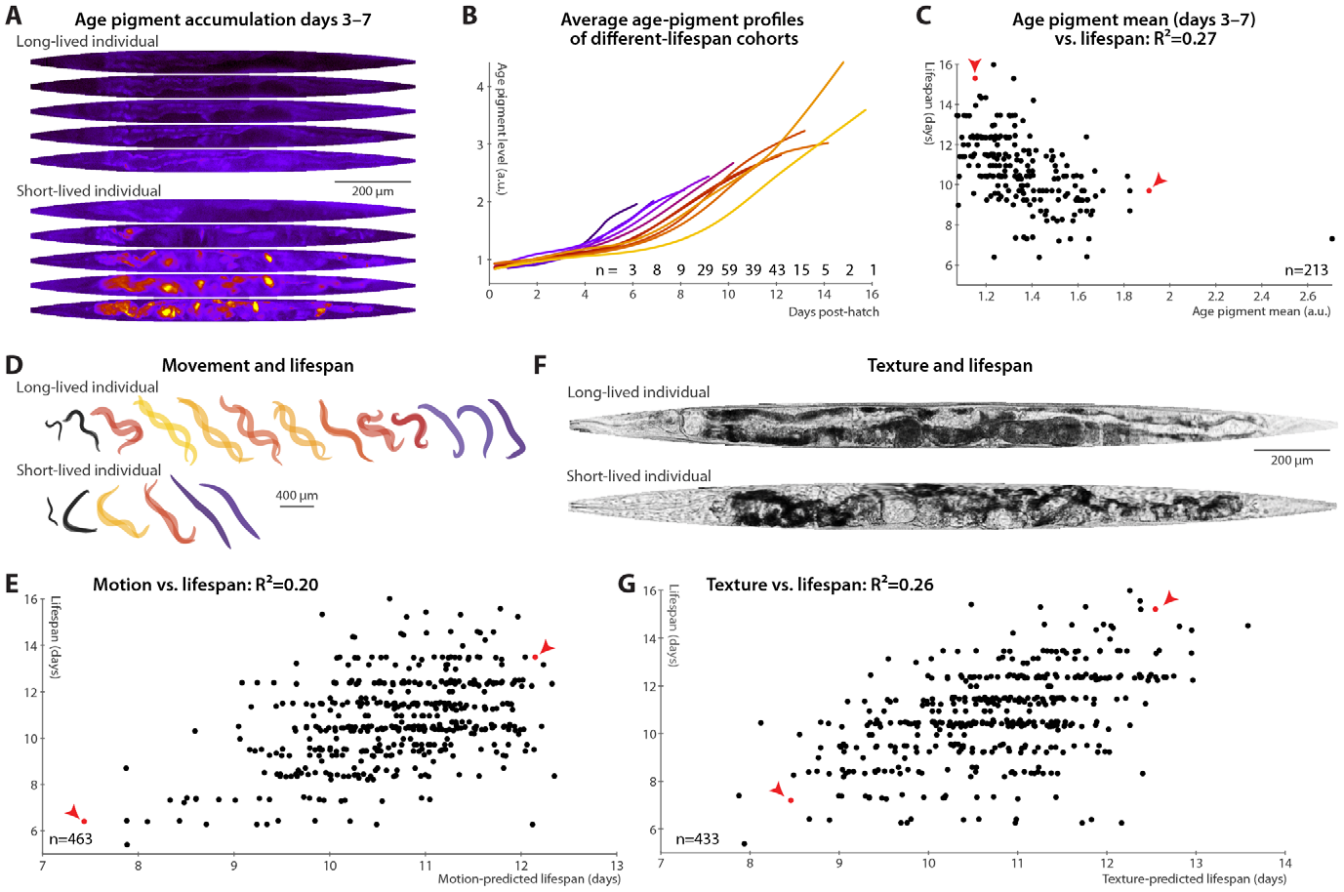
Phenomenological predictors of nematode longevity. (A) Autofluorescence images of two individual nematodes with different rates of age pigment accumulation at days 3–7 (top–bottom). Images were warped to the average day-5 shape and size for simple comparison and pseudocolored on a black-blue-red-yellow spectrum to provide sufficient dynamic range. (B) The average level of age pigment accumulation (measured as the 95^th^ percentile of whole-body autofluorescent intensity) over time is shown for cohorts grouped by lifespan. Shorter-lived animals in general have higher and faster-rising autofluorescence. (C) Average levels of age pigment, measured between days 3 and 7, anti-correlate with longevity; R^2^ = 0.27 (*p*<10^−15^; l.o.o. 0.25); the slope of autofluorescence accumulation, days 3–7 (as in B) correlates similarly well. Both parameters jointly regressed against lifespan yield an R^2^ of 0.31 (*p*<10^−16^; l.o.o. 0.28). Points corresponding to the individuals in panel A are red and marked with arrowheads. (D) Movement rates of a long-lived and a short-lived animal throughout their lives are illustrated at each day of life by showing, superimposed, the animal's position in two images acquired 0.5 seconds apart. From day 3 onward, these animals are colored according to the movement score (see text) on a black-blue-red-yellow spectrum. The longer-lived animal moves more, both qualitatively and quantitatively. (E) Regressing both the mean motion score between days 3 and 7 and the slope in that time range against each animal's lifespan yields an R^2^ of 0.20 (*p*<10^−22^; l.o.o. 0.19). The data points corresponding to the individuals in panel D in red and marked with arrowheads. (F) Straightened brightfield images of a texturally decrepit (bottom) and non-decrepit (top) individual, both 7 days post-hatch. (G) A “texture decrepitude” score is calculated daily (see text); the mean score between days 3 and 7 and over the slope in that time range jointly predict each individual's longevity with an R^2^ of 0.26 (*p*<10^−28^; l.o.o. 0.25). The data points corresponding to the individuals in panel F in red and marked with arrowheads.

It had been previously speculated that that age pigments, known to correlate with the current health state, will be predictive of future longevity [19], though this was not borne out in a recent study [20]. We therefore tested accumulation of autofluorescent age pigments (imaged through a red filter set and apparent in gut granules and in aged gonads; see Materials and Methods and Figure S4). Figure 2A shows the patterns in autofluorescent age pigment accumulation in two individuals, computationally straightened and fit to the average day-5 shape and size for visualization, between days 3 and 7 post-hatch; Figure 2B shows the pigment accumulation trends for cohorts with different lifespans. (These measurements are of the 95^th^ percentile of pixel intensity within the defined “worm region” of the original images; here and in all subsequent cases, other measures such as mean or median yield similar results.) Clearly, the longer-lived animals have lower absolute levels of pigmentation, even early in life, and also lower rates of increase in pigment levels. Prospectively, animals with higher levels of autofluorescence, and also those with higher rates of autofluorescence accumulation, in days 3–7 post hatch are likely to experience shorter lifespans (Figure 2C).

Various measures of movement rates have been shown to predict future longevity [11]–[14]; we attempted to replicate this finding by calculating a daily movement score from pixel intensity differences in sequential images (see Materials and Methods); higher scores indicate more movement (Figure 2D). We found that the mean and slope of the motion score, days 3–7, positively correlate with eventual longevity (Figure 2E). That is, high movement rates and maintenance of these rates through mid-adulthood are markers of longer lifespan, strengthening the conclusions from previous studies. Finally, aged *C. elegans* have a very typical “decrepit” appearance in brightfield images [11], [18]. As quantitative measurements of image texture have previously been used as proxies of age-related tissue deterioration (in particular, sarcopenia) in nematodes [44], [45], we defined a daily measure of whole-animal textural decrepitude (see Materials and Methods and Figure 2F) which, examined between days 3 and 7, predicts a sizable fraction of longevity variation (Figure 2G): more deteriorated-appearing animals (high mean decrepitude days 3–7) and those that more rapidly become so (positive slope) are shorter-lived.

Overall, each of the individual measurements shown in Figure 1 and Figure 2 consists of a phenomenological evaluation of one or more aspects of nematode health states, encompassing sarcopenia (motion, image texture, size), tissue maintenance (image texture, size), and autophagocytic ability (autofluorescence accumulation). Because these measurements of tissue and cellular homeostasis are integrative and relatively “downstream”, they provide powerful mid-life predictors of eventual longevity; however, they yield few clues regarding the origin of individual differences in longevity.

### *mir-71* Expression Levels and Spatial Patterns Predict Lifespan

**Figure 3:**
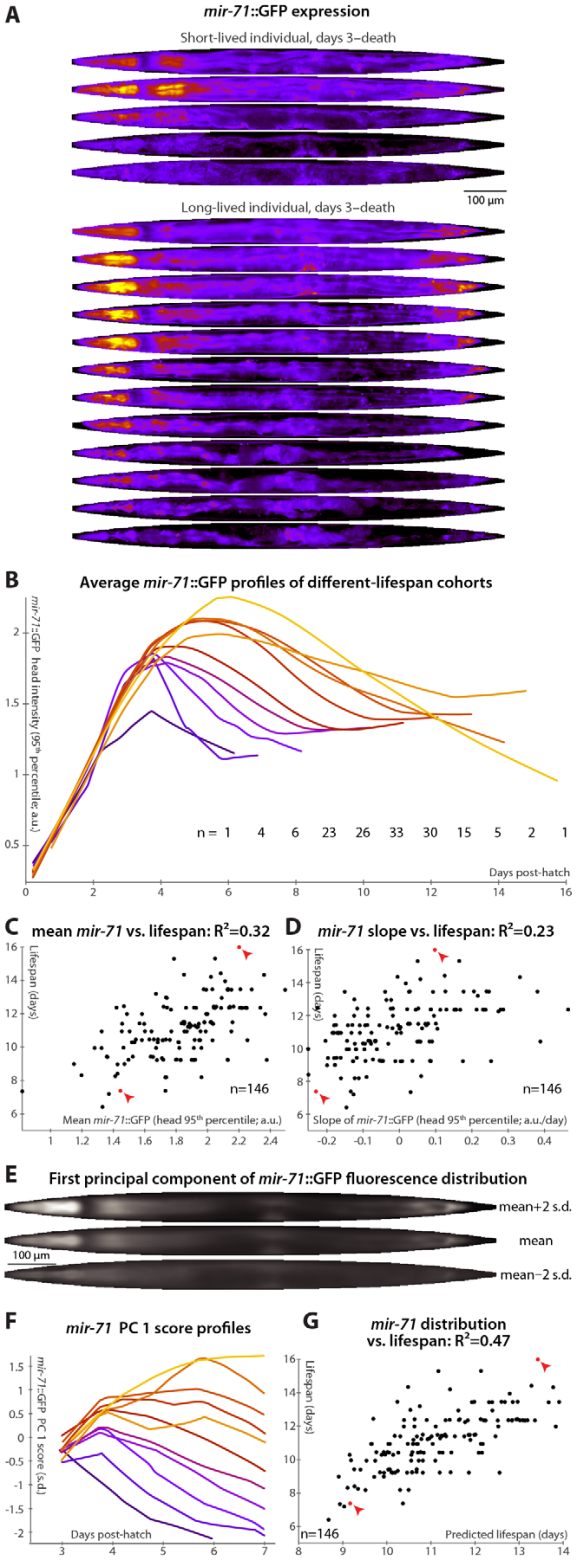
*mir-71*::GFP levels and expression patterns predict longevity. (A) Daily images of *mir-71*::GFP expression patterns for two individuals, from day 3 to the last day of life are shown (top–bottom), straightened and pseudocolored as in Figure 2A. (B) Average *mir-71*::GFP expression (measured as the 95^th^ percentile of head-region intensity) versus time is shown for cohorts with different longevities. Shorter-lived animals have, in general, lower and more rapidly declining levels of *mir-71*::GFP expression. (C,D) The mean of (C) and slope of a fit line to (D) days 3–7 *mir-71*::GFP expression correlates with each animal's future longevity. Regressing both jointly against longevity yields an R^2^ of 0.35 (*p*<10^−13^; l.o.o. 0.32). (E) The mean of 979 warped and aligned images of *mir-71*::GFP expression is shown, along with synthetic images illustrating two-standard-deviation offsets from that mean along the first principle component (PC1; the set of correlated changes in pixel intensities that together explain the maximal variance in the data set). In this case, PC1 spans 18% of the variability in the image data. This component reflects changes in the tissue-specificity of *mir-71*::GFP expression. An image can be scored in terms of standard deviations from the mean along this component (PC score); more positive scores indicate head/vulva/tail specificity and more negative scores indicate diffuse background expression. (F) Trends in PC scores (calculated only between days 3 and 7) are shown for the different-longevity cohorts in panel B. Higher scores and slowly falling scores are clearly associated with longer life. (G) The mean and fit slope of the PC scores of days 3–7 *mir-71*::GFP expression jointly predict future longevity; R^2^ = 0.47 (*p*<10^−19^; l.o.o. 0.45). The use of this prediction as a test for actual above-average longevity (including sensitivity and specificity figures) is shown in Figure S2D.

The miRNA *mir-71* increases rapidly in expression during larval development, peaks at early to mid-adulthood, and then gradually declines (Figure 3A, 3B and [36]). Beyond differences in lifespan and stress-resistance [36], *mir-71* mutant animals appear phenotypically wild-type [46]. Further, *mir-71* genetically interacts with IIS downstream of the insulin receptor homolog *daf-2* but upstream of *daf-16*, the FOXO transcription factor that is a major IIS effector [36]. miR-71 is predicted to target several genes in the IIS pathway; of these, *pdk-1* levels are greatly increased in aged animals lacking *mir-71* [36]. Additionally, *mir-71* appears to be both a downstream target of and a regulator of DNA damage responses via CDC-25.1 [36]. A transgenic reporter, *mir-71*::GFP, containing the promoter of *mir-71* driving GFP expression, was previously characterized [36], [47]. Though ubiquitously expressed during adulthood, *mir-71*::GFP expression is most prominent in the hypodermis, pharynx, vulva, intestinal, and tail cells [36], [47], [48].

Longer-lived cohorts have distinctly different temporal patterns of *mir-71*::GFP expression compared to shorter-lived siblings (Figure 3B). Expression levels are here quantified as the 95^th^ percentile of pixel intensities in the worm's head region; other measurements (mean, median, etc.), and/or aggregating across the whole body, produce similar results though we find this to be the most robust. Specifically, retention of “youthful” *mir-71* states, both in terms of high levels of *mir-71*::GFP expression and of maintenance of these levels through mid-adulthood, correlates with longevity. Prospectively, both the mean *mir-71*::GFP expression levels and the change in these levels between days 3 and 7 both correlate with future lifespan variability (Figure 3C and 3D); together these two parameters predict 35% of lifespan variation (the l.o.o. value is 32%). Animals with higher or longer-lasting *mir-71*::GFP expression tend to live longer, consistent with the known role of miR-71 in promoting lifespan [36].

To further examine and quantify trends in *mir-71*::GFP expression patterns, we used principal components analysis (PCA). This procedure is conceptually similar to one described recently [49], which employed hierarchical clustering instead of PCA. We used 979 fluorescent images from 146 individual animals, controlled for individual differences in size, shape, internal compression due to locomotion, and overall *mir-71*::GFP expression level (see Materials and Methods) so that the analysis captured trends only in the spatial pattern of expression. The first principal component, which by definition explains the single largest correlated trend in the dataset (18% of total expression-pattern variability in this case), captures a transition from a highly specific head/vulva/tail expression pattern to more diffuse whole-body expression (Figure 3E). As the individuals shown in Figure 3A illustrate, there is both low-level whole-body background expression of *mir-71*::GFP that remains relatively constant over time, and strong head/vulva/tail-specific expression that peaks and declines. The position of the *mir-71*::GFP expression pattern along this principal component (“PC score”), in terms of standard deviations above or below the mean expression pattern (Figure 3E), therefore quantifies the degree of strong, tissue-specific expression at a given day. Figure 3F clearly shows that longer-lived cohorts have more positive and increasing scores, corresponding to high (and increasing) degrees tissue-specificity of expression, while short-lived cohorts have more negative and decreasing scores. Quantitatively, maintenance of head/vulva/tail expression (measured here by the slope of days 3–7 PC scores, though in general other approaches could be employed), and the average overall degree of head/vulva/tail expression (mean PC score day 3–7), are highly correlated with longevity (R^2^ = 29% for slope and 39% for mean); jointly they predict 47% of individual longevity variation (Figure 3G; l.o.o. 45%). Animals with above-average predicted longevities based on these two measurements have substantially different observed lifespans than those with below-average predictions, illustrating the utility of these measures as a diagnostic test of longevity (Figure S2D; the difference in mean lifespan between these two cohorts is 20%).

We confirmed that GFP expression alone does not predict lifespan by examining animals bearing the *mIs10* transgene, which contains a *myo-2* promoter driving GFP expression in the larval and adult head, a gut enhancer driving intestinal GFP expression in the adult, and *pes-10*::GFP, which is expressed embryonically. We found no whole-body or head-only summary of GFP (mean/median/95^th^ percentile/etc.) that, measured in terms of mean or slope over days 3–7 (or various other ranges), predicts longevity to any significant or substantial degree in this dataset (not shown). In addition, other miRNA promoter::GFP fusions correlate (and anti-correlate) with longevity to different degrees (below).

**Figure 4:**
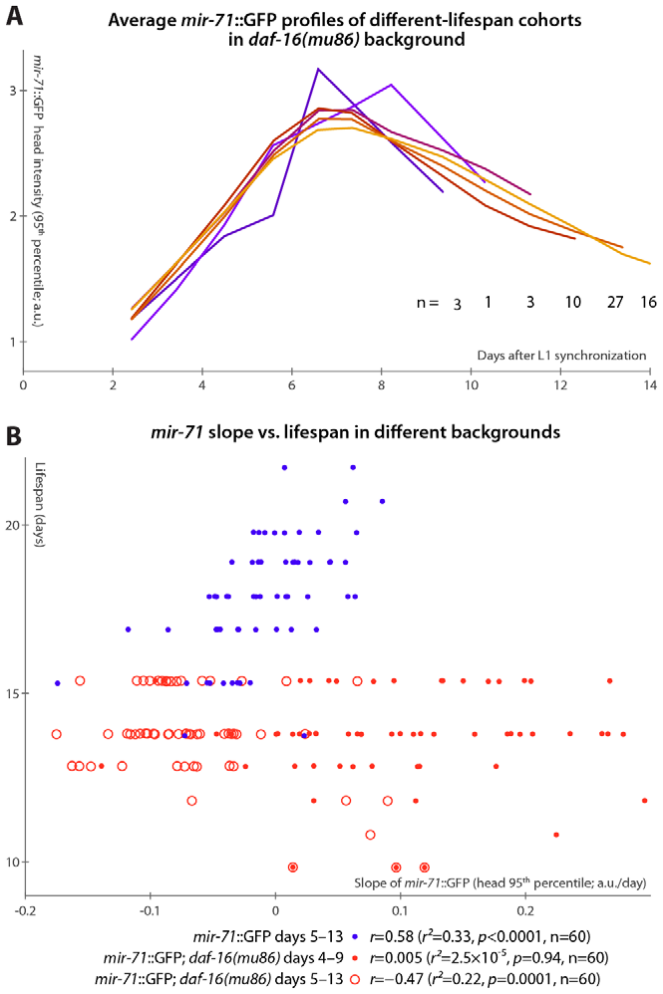
*mir-71*::GFP levels do not positively correlate with longevity absent DAF-16. (A) Average *mir-71*::GFP expression (measured as the 95^th^ percentile of head-region intensity) versus time is shown for cohorts of *mir-71*::GFP; *daf-16(mu86)* animals with different longevities. In contrast to Figure 3B, without DAF-16, longer-lived cohorts are not clearly distinct from shorter-lived cohorts in terms of temporal trends in *mir-71*::GFP expression. (B) The relationship between the slope of *mir-71*::GFP expression in the head in a defined time window and ultimate lifespan is shown for various strains and time windows. Blue circles mark *mir-71*::GFP animals, measured days 5–13 (which corresponds to the day 3–7 window after adjusting for the different lifespan induced by the culture conditions; see Materials and Methods and Figure S5A). Closed red circles mark *mir-71*::GFP; *daf-16(mu86)* animals measured days 4–9 (an adjustment accounting for the shortened lifespan of the strain), while open red circles mark the same strain measured days 5–15. In all cases, the reported correlation values do not strongly depend on the starting day.

If the primary lifespan-determining target of miR-71 regulation is IIS, then *mir-71*::GFP expression patterns should no longer correlate with lifespan absent IIS. We tested this by examining *mir-71*::GFP fluorescence in a *daf-16(mu86)* background, which lacks this primary IIS effector. Because *daf-16* lies extremely close to the *spe-9* genomic locus, it was impractical to construct a *mir-71*::GFP; *daf-16*; *spe-9* strain. Thus, we modified our experimental protocol to use and to allow for the placement of synchronized young-adult animals onto gel pads treated with the drug 5-fluoro-2′-deoxyuridine (FUDR) to prevent reproduction in the culture apparatus (see Materials and Methods). In this regime, lifespan was somewhat extended, along with a concomitant “stretching out” of the rise-peak-fall temporal expression pattern of *mir-71*::GFP intensity (Figure S5). This may be at least partially due to the FUDR, which has been shown to extend lifespan in an environment-dependent fashion [50]. Nevertheless, *mir-71*::GFP levels remain predictive of longevity after the day 3–7 measurement window is adjusted to account for the lifespan extension (Figure S5A and Figure 4B).

However, in the *daf-16(mu86)* background, *mir-71*::GFP expression differences were no longer apparent between cohorts with different lifespans (Figure 4A). Because *daf-16(mu86)* animals are short-lived, we adjusted the “early adulthood” window in which measurements of GFP expression were made to match (Figure S5A); in this window, neither the increase in (Figure 4B), nor the mean level of (not shown), *mir-71*::GFP positively correlated with lifespan. If no accounting for shortened lifespan is made (and, indeed, we observe no compression of the rise-peak-fall temporal expression pattern of *mir-71*::GFP in the *daf-16* background; Figure S5B), we found that GFP expression changes anti-correlate with lifespan (Figure 4B). This anti-correlation appears to be driven by a small subpopulation of the most short-lived animals which have very high *mir-71*::GFP expression levels. Thus, in a *daf-16* null background, the predictive power of *mir-71*::GFP expression is either suppressed, or, potentially, reversed.

Finally, we note that compared to matched *mir-71*::GFP controls, *mir-71*::GFP; *daf-16(mu86)* animals had approximately double the peak GFP expression levels (Figure S5B), though the shape of the temporal pattern (Figure S5B) and spatial distribution (Figure S5C) of expression remained extremely similar. This may suggest negative-feedback regulation of miR-71 by DAF-16 or one of its targets.

### Rates of *mir-246*/*mir-239* Increase Promote/Antagonize Individual Longevity

**Figure 5:**
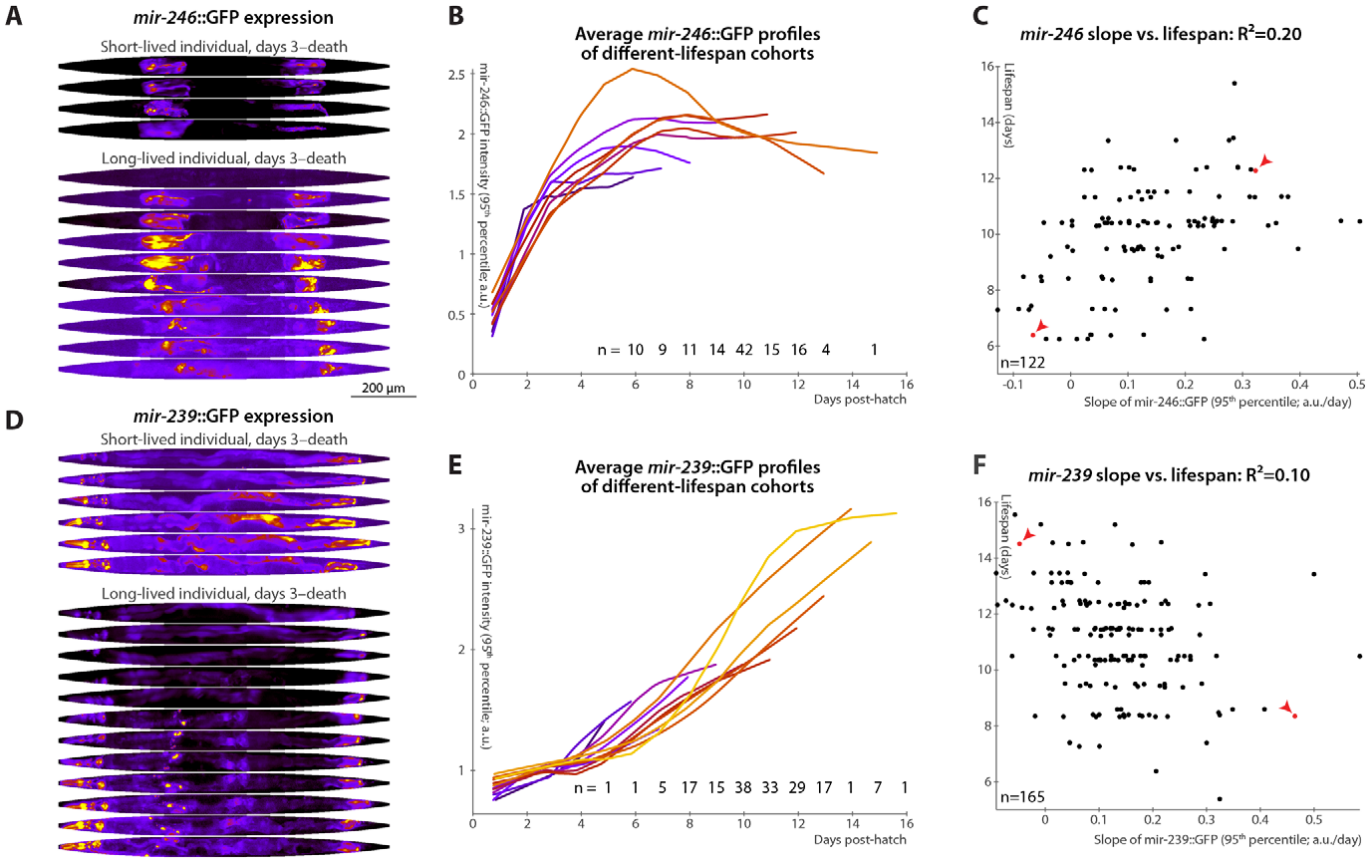
Changes in *mir-246*::GFP and *mir-239*::GFP expression over time predict longevity. (A,D) Daily images of *mir-246*::GFP (A) or *mir-239*::GFP (D) expression patterns for two individuals, from day 3 to the last day of life are shown (top-to-bottom), straightened and pseudocolored as in Figure 2A. (B,E) Average *mir-246*::GFP (B) or *mir-239*::GFP (E) expression (measured as the 95^th^ percentile of whole-body intensity) versus time is shown for cohorts with different longevities. Shorter-lived animals have, in general, more rapidly declining levels of *mir-246*::GFP expression and slightly more rapidly increasing levels of *mir-239*::GFP. (C) The slope of a fit line to *mir-246*::GFP intensity between days 3 and 7 correlates with each animal's future longevity; R^2^ = 0.20 (*p*<10^−6^; l.o.o. 0.18). Points corresponding to the individuals shown in panel A are in red and marked with arrowheads. (F) The slope of *mir-239*::GFP, days 3–7, anti-correlates with future longevity; R^2^ = 0.10 (*p*<10^−4^; l.o.o. 0.070). Individuals from panel D are in red and marked with arrowheads. The use of the predictions from panels C and F as tests for actual above-average longevity (including sensitivity and specificity figures) are shown in Figure S2E and S2F.

Much like *mir-71*, *mir-246* mutants appear phenotypically wild-type except for decreased longevity and stress-resistance [36], [46]. The expression of miR-246 increases over time, and a *mir-246*::GFP construct shows that the gene is expressed in the gonadal sheath [36], [47]. Our detailed analysis of *mir-246*::GFP in individual animals shows a gradual plateauing of *mir-246* expression in late adulthood (Figure 5A and 5B), but, unlike *mir-71*, no concomitant loss of tissue specificity. We find that animals in which *mir-246*::GFP levels plateau more slowly (measured by the slope of *mir-246*::GFP 95^th^-percentile fluorescence intensity between days 3 and 7) are relatively longer-lived: change in *mir-246* expression in this time range predicts 20% of total longevity variation (l.o.o. 18%; Figure 5B and 5C). The mean level of *mir-246*::GFP between days 3 and 7, however, does not clearly predict longevity. Additionally, we observed that while the distributions of lifespans for animals with slow- vs. fast-increasing *mir-246*::GFP expression are significantly different (Figure S2E), they have nearly identical modal values; however, a subset with particularly early mortality appears to be associated with low *mir-246*::GFP slopes. None of the principal components of spatial expression variability correlate with longevity: *mir-246*::GFP expression is highly tissue-specific, and so the principal components predominantly capture uninteresting variations in the internal position of the gonad sheath.

Unlike *mir-71* and *mir-246*, *mir-239* antagonizes longevity: mutants lacking the identical *mir-239a* and *mir-239b* sequences have increased lifespan and stress-resistance (though, again, no other clear phenotypes) [36], [46]. Genetic experiments suggest that *mir-239* is downstream of *daf-2* and upstream of *daf-16*; miR-239 may promote IIS (which is anti-longevity) via indirectly increasing levels of the cytoplasmic IIS transduction components AGE-1 (the catalytic subunit of phosphatidylinositol 3-kinase) and PDK-1. *mir-239*::GFP expression is predominantly in several head and tail neurons, with lower levels in pharyngeal and gut tissues [36]. While *mir-71* and *mir-246* levels peak or plateau, respectively, at mid-life (Figure 3A, 3B and Figure 5A, 5B), we observe that *mir-239*::GFP expression levels drift upward over time (Figure 5D and 5E). Consistent with its role as a lifespan antagonist, higher *mir-239*::GFP levels correlate with shorter lifespans. As there is relatively little variability in *mir-239*::GFP expression at post-hatch day 3, the days 3–7 increase and the day-7 magnitude of expression capture similar information, and both predict approximately 10% of longevity variability. For the slope measure, the l.o.o. R^2^ is 7% (Figure 5F). Though this value is somewhat low compared to the others reported, the lifespans of high and low *mir-239*-expressing animals remain significantly and substantially different, with a difference in means of approximately one day (Figure S2F), or ∼10% of the average lifespan; moreover, as a diagnostic test for above-average longevity, above-average *mir-239*::GFP slope performs nearly as well as *mir-246*::GFP or the motion- or texture-based measures (Figure S2). As with *mir-246*::GFP, PCA applied to *mir-239*::GFP images did not yield any expression-pattern trends predictive of future longevity.

### Relationships among Longevity Biomarkers

**Figure 6:**
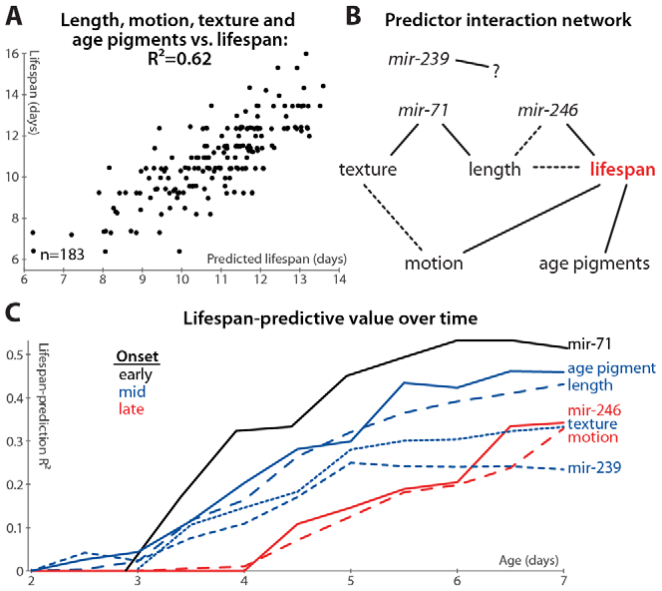
Multivariate lifespan predictions and relationships between biomarkers. (A) Multivariate regression of length (days 3–7 mean and slope of fit line), motion (mean and slope), texture decrepitude (mean and slope), and autofluorescence accumulation (slope) against lifespan yields a predicted lifespan or “survival index” that explains 62% of variability in future longevity. (*p*<10^−32^; l.o.o. estimate 57%; see also Figure S2G). (B) A partial correlation network illustrates the pattern of conditional independences between measured parameters, which are directly connected if and only if they correlate with one another after controlling for all subsets of other parameters. The network shown is a consensus from several datasets (see Figure S6); dashed lines indicate relations that are not fully consistent, and *mir-239* cannot be placed into the network at all. (C) Lifespan-predictive ability of each biomarker as a function of age. R^2^ values from regressing lifespan against biomarker measurements up to a given age are plotted versus that age. (Texture, motion, and *mir-71*::GFP PCA measures were calculated only from day 3 onward.)

We measured many of the reported biomarkers in the same animals, enabling us to construct a multivariate predictor of nematode longevity, which we term the “survival prediction index”. This model, incorporating the length, motion, texture, and autofluorescence-accumulation measurements described in Figure 1 and Figure 2, predicts 62% of all lifespan variability across all datasets for which these parameters were measured (l.o.o. 57%; Figure 6A), and above-average “survival prediction indices” divide the animals into well-delineated long-lived and short-lived subgroups (Figure S2G; mean lifespan is 22% increased in the high-survival-index cohort compared to the low-index cohort). The relative importance of each biomarker can be inferred by examination of the relative regression weights: autofluorescence (slope): −0.302; length (slope): 0.266; length (mean): 0.174; motion (mean): 0.149; motion (slope): 0.137; texture (mean): 0.121; texture (slope): 0.077. (To render regression weights directly comparable, all input parameter values were expressed in unit-free terms of standard deviations from their mean; including mean autofluorescence values did not improve lifespan-predictive ability.) Overall, the measures of size and age pigments dominate (for these measures alone, lifespan-prediction R^2^ = 55%, l.o.o. 53%).

Further, we find that adding *mir-71* or *mir-239* measurements to those above do not improve the lifespan predictions of the model (not shown), indicating that these measurements provide information that is also captured by the downstream, phenomenological markers (see also below). However, adding *mir-246* slope measurements adds at least 5% to the R^2^ value attained from the phenomenological markers, which suggests that miR-246 promotes longevity via more than just the mechanisms reported on by length, texture, motion, and autofluorescence.

We formalized this analysis by inferring the conditional independencies of various parameters from partial correlations. That is, while all of the biomarkers correlate with one another, it is possible to statistically infer whether these relationships are direct or indirect. For example: *mir-*71 levels correlate with longevity, but this correlation is largely abrogated when length maintenance is controlled for; conversely, controlling for *mir-71* reduces the correlation between length and lifespan to a much lesser degree (see Table S2, which lists the correlation of each marker with longevity after various controls). Therefore either *mir-71* influences length, which then influences longevity, or length variability is an upstream cause of both *mir-71* variability and lifespan variability. To systematically evaluate these interactions, we constructed a partial correlation network (also known as a graphical Gaussian model) from our data (Figure 6B; see Materials and Methods) [51]. We have not yet directly evaluated the relationship between the different miRNAs, which requires measuring the promoter activity of these genes in the same animals. We can, however, indirectly infer the relationship between these miRNAs via intervening factors measured in all datasets: the network in Figure 6B reflects the consensus of networks calculated from each dataset (Figure S6). While the relatively weak relationship between *mir-239* and lifespan prevents its accurate placement into the network, other trends are clear. Age-pigment accumulation, motion, and texture/*mir-71*/length provide relatively independent information about lifespan from one another, though there may be some relation between the motion and texture scores (Figure S6). Including *mir-246* decreases the link between length and lifespan (Figure S6 and Table S2), suggesting that, while its precise position is indeterminate, *mir-246* is either an upstream cause of both length and lifespan variation, or *mir-246* levels are determined by physiological processes that regulate length maintenance, and these levels more directly control lifespan.

Finally, we examined the timing of each measure's lifespan-predictive ability: do some measurements provide earlier hints at longevity than others? The accuracy of lifespan prediction for each measure (measured as the R^2^ value) is plotted versus time in Figure 6C. For this analysis, we did not manually determine one or two summaries of the time-course of biomarker measurements (e.g. “mean length between days 3 and 7”), but instead predicted longevity using all of the raw time-course data up to a given age (e.g. to evaluate the predictive ability of length up to day 4, we regressed lifespan against the lengths measured at days 2, 3, and 4). We used ridge-regression with an automatically-determined penalty in order to prevent over-fitting due to the increased number of parameters at later time-points (see Materials and Methods); note however that the R^2^ values are in some cases more optimistic compared to the simpler measurements defined earlier.

Based on this analysis, we observe that the various predictors of longevity differ markedly in their timing: the *mir-71* PCA scores provide the earliest, and best, predictors of lifespan, while *mir-246* and motion provide the latest-onset information about future lifespan.

## Discussion

We used a novel culture system that allowed us to quantitatively examine individual nematodes throughout time and directly correlate inter-individual variability in early life with variability in eventual lifespan. Through this observational study, we identified phenotypic markers that, measured while >97% of the population remains alive, predict over 60% of future individual longevity variability. In addition to confirming markers that previously had been shown to correlate with lifespan (movement rates [12]–[14]) and widely suspected to do so (visual decrepitude [11], [18], [45], age pigment accumulation [17]–[19], [43]) we have also found a novel biomarker of longevity in maintenance of adult size through mid-life (Figure 1E and 1F). This dovetails with the recent finding that juvenile “growth span” (time to reach adult size) also predicts lifespan [14].

Our results regarding age pigmentation are inconsistent with others recently reported [20], an observation which may be explained by the fact that we measured age pigmentation in the red spectral range (see Materials and Methods), while Sánchez-Blanco and Kim examined green autofluorescence. Note that lipofuscin, *per se* (as opposed to other autofluorescent “age pigments”), fluoresces most strongly in blue ranges [21]. This indicates the potential heterogeneity of age pigmentation across the color spectrum.

Seeking a more detailed understanding of the genetics of individual longevity determination, we found that fluctuations in the levels of miR-71, 239, and 246 (imputed via promoter::GFP reporters) predict a substantial portion of longevity. To date, only one other group has reported any genes, in any species, in which endogenous, un-perturbed fluctuations in expression level have been reported to be predictive of future longevity [20]. The ability of expression states to predict later longevity quite early in life further suggests that some fraction of longevity variance is indeed the result of developmentally determined epigenetic states of “robustness” or “frailty”. This hypothesis has been raised theoretically [52], and the degree of post-heat-shock longevity-extension (correlated with HSP-16.2 expression [25]) has been shown to fit this model well [53], [54].

In particular, the degree of strong, tissue-specific *mir-71* expression in the head, vulva, and tail is the single most powerful biomarker identified, explaining 47% of future longevity (Figure 3F and 3G), and also the earliest indicator of future longevity (Figure 6C). Combining these findings, which are based on observation of essentially unperturbed individuals, with earlier knockout studies that demonstrate that abrogation of these miRNAs alters mean lifespan [36], we infer that individual, wild-type variability in the expression of these regulators not only reports on longevity but may likely determine it as well. (In all cases, the correlation or anti-correlation of miRNA levels with longevity is in the same direction as suggested by previous knockout and overexpression studies [36].) Note that it is not necessarily the case that genes that alter lifespan when removed or artificially overexpressed will determine individual lifespan in wild-type conditions: core health-determining genes may be tightly regulated to have low inter-individual variation, or that variability may be buffered downstream and not translate into altered longevity.

In the case of *mir-71* and *mir-239*, the mechanism of individual lifespan determination may be via the well-known insulin/IGF-1-like signaling pathway (IIS) [36], the absence of which promotes longevity via stress-response and macromolecular homeostatic mechanisms, among others [55]–[57]. In particular, *mir-71* knockout led to increased expression of components of the IIS transduction machinery [36], and we here found that *daf-16* null animals have increased levels of *mir-71*::GFP expression. These observations suggest that DAF-16 and miR-71 levels are held in homeostatic balance via a mutually regulatory feedback loop. Thus, it is possible that inter-individual fluctuations in miR-71 levels may directly determine inter-individual levels of tonic IIS activity, and hence, individual lifespan. This conclusion is strengthened by our finding that, absent DAF-16, *mir-71*::GFP is no longer predictive of longevity. One final piece of evidence for the role of IIS in lifespan determination comes in the work of Sánchez-Blanco and Kim [20], which identifies *daf-16* and *sod-3*, a canonical DAF-16 target, as among the best predictors of individual longevity. (In fact, *sod-3* reporter expression is their strongest predictor reported, with an R^2^-value of 0.32.)

Further, *mir-71* may also act by downregulating cell-cycle checkpoint proteins [36], the absence of which also promotes stress-response factors, even in postmitotic cells [58]. The early predictive ability of *mir-71*::GFP suggests that *mir-71*, and thus either or both of the signaling pathways it regulates, may play an early-life role in determining organismal robustness and longevity.

According to our network analysis, image texture, *mir-71*::GFP expression, and size are all closely related, with size as the most downstream measure of what we take to be the age-regulated tissue disorganization and sarcopenia reported previously [11], [18]. Overall, and especially given *mir-71*'s role in regulating pathways that determine levels of stress responses, we suspect that this nexus of predictors reflects processes of somatic growth and maintenance. However, while both *mir-71* and *mir-246* levels are correlated with length maintenance, *mir-246*, unlike *mir-71*, provides additional information about longevity not captured by length or other “somatic maintenance” features. Given this, the relatively late (i.e. post-reproductive) timing of its predictive ability, the sharp upregulation of *mir-246*::GFP expression at reproductive maturity (Figure 5A, 5B and [47]), and its localization literally at the interface between the gonad and other somatic tissues, it is tempting to speculate that *mir-246* is involved in balancing reproduction and somatic maintenance [32], [59]. Further, we find that age pigment accumulation also provides a degree of information about lifespan not captured by the “somatic maintenance” nexus, suggesting that it, too, reflects an inter-related but parallel lifespan-determining process.

It is also worth examining these results in light of those of our previous work, which compared miRNA expression over time in wild-type and long-lived *daf-2* IIS mutant animals [36]. Often, *daf-2* animals are taken as a paradigm case of “long-lived” animals, and the presence or absence of a physiological feature in these mutants assumed indicative of a role as a biomarker of successful aging. While in many cases this is undoubtedly so (for example, decreases in rates of lipofuscin accumulation in these animals [19]), our results yield the unsurprising finding that the relationship between physiological events in *daf-2* mutants and long-lived wild-type animals are inexact. Specifically, we here find clear evidence that elevated levels of *mir-71* and *mir-246* expression are associated with extended longevity, yet in *daf-2* animals these miRNAs are not upregulated, likely reflecting their upstream, negative-regulatory roles in insulin signaling [36]. Similarly, we find that *daf-16* animals are physiologically quite dissimilar from short-lived wild-type individuals in terms of *mir-71*::GFP expression. Intact individuals which are short-lived typically have low *mir-71*::GFP levels, while *daf-16* animals show a dramatic elevation in *mir-71*::GFP. Mechanistically, this may be due to disrupted negative feedback from DAF-16 (or a target) on *mir-71*; pragmatically it suggests the limitations of inference about the physiology of intact animals based on findings in particular mutants. In this case, based only on the average difference in *mir-71*::GFP in *daf-16* vs. wild-type, one might incorrectly conclude that high miR-71 levels are a marker of short lifespan. We thus believe there is great utility to the quantitative observation of individual wild-type (or nearly so) animals.

Lastly, in all cases observed, it is the retention of young-adult-like trends (high and/or increasing length, *mir-71* and *246* expression; low or slowly-increasing autofluorescence and *mir-239*) into mid- and late-adulthood that predicts longer lifespan. Classic antagonistic pleiotropy theories of aging posit that some age-related degeneration may be due to alleles which are beneficial in early life but become damaging over time (“live fast, die young” effects) [60]; however, in the examples here presented we find that the loss of youthful biometrics, not their continuation, proves most harmful.

Thus, from this and previous studies [36], these miRNAs appear to be relatively upstream regulators of lifespan-determining pathways that are relevant to the determination of inter-individual variation in nematode lifespans. The miRNAs we have identified are not well conserved in higher animals so these particular mechanisms of lifespan determination are likely nematode-specific. However, given the conserved nature of aging pathways across phylogeny, our work does suggest that fluctuations in other regulators of these pathways (including other miRNAs) may predict or determine individual aging rates in more complex organisms, perhaps foreshadowing or even controlling the timing of age-related decline in humans.

## Materials and Methods

### Single-Animal Vermiculture

Waterjet-cut borosilicate glass slides were obtained from Advanced Waterjet and Engraving (Anaheim, CA). Before and after use, slides were soaked in a base bath (2:5 isopropanol:H_2_O, 1.5M KOH) to remove all organic material. Prior to use, the slides were treated to functionalize the glass surface with reactive methacryl groups as follows: slides were rinsed in distilled H_2_O (dH_2_O) and submerged in 5% HCl (aq.) for 10 min to protonate surface hydroxyls, rinsed again in dH_2_O and then submerged with agitation for 2 min. in methacryl silane solution (2% ^v^/_v_ 3-methacryloxypropyltrimethoxysilane [Z-6030, Dow-Corning; Midland MI] in 95% ethanol with 0.02% ^v^/_v_ glacial acetic acid, made fresh and stirred vigorously for 10 min. immediately prior to use). The slides were then rinsed in 95% ethanol, heated at 110°C to effect the condensation of the silane reagent to the glass surface, and stored with desiccant. Prior to use, one side of each slide was sealed with Scotch Premium Performance packing tape (3M; St. Paul, MN).

We use a methacryl-difunctional polyethylene glycol to create a crosslinked hydrogel [61] that, when polymerized in the methacryl-derivatized glass wells, crosslinks also to the sides of the wells. This prevents the high rate of escape down the sides of the wells that we observe when using agar gels. Agar-free but otherwise standard nematode growth media [62] was supplemented with 4% ^w^/_v_ dimethacryl PEG-1000 (Polysciences; Warrington, PA), 4% ^w^/_v_ monomethacryl PEG-1100 (Sigma-Aldrich; St. Louis, MO) as a plasticizer, and 0.1% ^w^/_v_ 1-[4-(2-hydroxyethoxy)-phenyl]-2-hydroxy-2-methyl-1-propane-1-one (Irgacure 2959, BASF; Ludwigshafen, Germany), a water-soluble, photo-activatable crosslinking initiator. This PEG-NGM was filter-sterilized and pipetted to fill the wells in the glass slides level to the top. The slides were then placed in a sealed chamber with a UV-transparent borosilicate glass lid, which was purged with nitrogen and exposed to 1.5 J of shortwave UV (λ_max_ = 350nm) radiation to initiate crosslinking.

1 µL 12.5% ^w^/_v_ *E. coli* OP50 (resuspended in M9) was pipetted onto each NGM-PEG pad, and individual eggs at the pre-hatch “pretzel” stage of development were transferred with an eyelash pick. Liquid polydimethylsiloxane (PDMS; Sylgard 184, Dow-Corning; Midland MI) was mixed 1∶10 with its crosslinking agent, de-gassed for 20 min. under vacuum, and pipetted atop the slide assemblies, which were then placed in 10 cm diameter polystyrene Petri dishes alongside small dH_2_O-saturated cotton strips (to prevent desiccation), sealed with parafilm, and stored at 23°C. PDMS polymerizes after approximately 12 hours in these conditions.

Most eggs hatch within 5 hours of slide preparation and reach their full adult size approximately 50 hours later. Ages reported are hours and days post slide preparation. These values are within the described range for this temperature [63], suggesting that our culture apparatus is substantially similar to standard conditions. Further, our observed mean lifespan of 10.7 days at 23°C (Figure 1B) is similar to our own measurements of *spe-9(hc88)* animals picked as pretzel-stage eggs onto standard NGM plates seeded with OP50 (mean lifespan = 9.5 days at 24°C, *n* = 350), and measurements of wild-type animals on solid media reported by others [6], [17]. However, we observe a somewhat smaller standard deviation in lifespan of ≈1.9 days vs. the 3–4 in those previous studies, suggesting that this culture apparatus provides an extremely uniform environment.

For the *daf-16* epistasis analysis, we modified the above protocol to allow for chemical sterilization of young adult animals by 5-fluoro-2′-deoxyuridine (FUDR; Sigma-Aldrich; St. Louis, MO). Specifically, FUDR from a 10 mg/mL aqueous stock was added at 1∶100 to PEG-NGM prior to filter-sterilization, which was polymerized in the glass slides as above. FUDR causes growth arrest of animals prior to the 4^th^ larval stage, so synchronized young adult animals were produced by hypochlorite treatment of gravid adults to isolate eggs [42] followed by overnight starvation in M9 buffer to synchronize animals as L1s, which were then plated on standard NGM-agar plates with OP50 food and allowed to grow to young adulthood at 23°C. These animals were transferred individually to PEG-NGM-FUDR slides supplemented with concentrated OP50 as above. Moving animals crawl into polymerizing PDMS, so the slides were sealed with 0.5mm-thick strips of PDMS that had been pre-cured on a glass plate at 100°C for one hour and cut to size. We observed an increase in longevity under these conditions relative to non-FUDR treated animals plated as embryos (Figure S5A).

### Strains

The following *C. elegans* strains provided by Caenorhabditis Genetics Center (CGC) were used in our studies: VT2084 (*mir-71*::GFP), VT1607 (*mir-246*::GFP), and PD4793 (mIs10: *myo-2*::GFP; *pes-10*::GFP; F22B7.9::GFP). *mir-239*::GFP was generated previously [36]. All strains were crossed into BA671, a *spe-9(hc88)* temperature-sensitive fertilization-deficient mutant, and assays were conducted at the restrictive temperature of 23°C. This strain has normal longevity at this temperature [42]. The small fraction of animals that reproduced in the culture apparatus and could not be clearly distinguished from their offspring was excluded from further analysis.

We determined that VT2084 actually contains the complete precursor miRNA sequence for miR-71 inside the “promoter” region driving transgenic GFP expression; therefore we wished to determine whether this strain overexpresses miR-71, which was previously shown to increase longevity [36]. Overexpression of miR-71 was not likely because the transgene in strain VT2084 was integrated via low-copy bombardment; however we confirmed via quantitative RT-PCR analysis (as performed previously [36]) that mature miR-71 levels in *mir-71*::GFP; *spe-9(hc88)* animals reared at 23°C (as similar as possible to our own culture conditions) showed minimal overexpression. Specifically, synchronized populations of *spe-9(hc88)* animals and *mir-71*::GFP; *spe-9(hc88)* animals were prepared by hypochlorite treatment and overnight starvation in M9, reared on NGM plates seeded with OP50 at 23°C, and harvested at 5 days post-plating, corresponding to the time of peak *mir-71*::GFP expression at young adulthood. Levels of miR-71 in *mir-71*::GFP; *spe-9(hc88)* animals were 102% or 115% that of *spe-9(hc88)* animals, depending on whether the U18 RNA or miR-66 (which are not temporally regulated), respectively, was used as a loading control. Mature miR-71 levels were also measured in homozygotic *mir-71* null *(n4115)*; *mir-71*::GFP animals prepared similarly; here, miR-71 expression was approximately 58% or 25% that of wild-type (using the U18 or miR-66 control, respectively). (The *n4115* strain without the *mir-71*::GFP transgene had undetectable miR-71 expression.) Further, we see no phenotypic consequence of the extra copies of the miR-71 sequence in VT2084: the lifespan of *mir-71*::GFP; *spe-9* animals in our apparatus is approximately 1.05 times that of the mean lifespan of the other strains analyzed, which is well within the range of inter-replicate variability. Thus, we conclude that for the purposes of this work, *mir-71*::GFP acts as a phenotypically wild-type reporter of miR-71 expression.

### Image Acquisition

We calibrated our microscope daily to control for spatial and temporal variation in light-source intensity, as described previously [36]. At the desired sample interval (typically daily), each slide was briefly removed from its humid chamber and placed in an upright microscope (Axioplan 2i; Carl Zeiss; Oberkochen, Germany), driven by custom software, for acquisition of brightfield and fluorescent images at 10× magnification. Per-slide acquisition time was typically under 20 minutes. Each animal was manually located and brought into focus, and a series of 10ms-exposure brightfield images were acquired, interleaved with fluorescence exposures of 1, 10, and 100 ms, in order to ensure that a properly exposed image was obtained. This sequence was performed for each filter-set of interest; in this case a GFP-bandpass filter to measure transgene expression (41017; Chroma; Bellows Falls, VT) and a TRITC filter to measure autofluorescence (41002c; Chroma).

While the peak autofluorescence of lipofuscin, a chief age pigment, is in the blue range [19], blue light evokes a strong escape response [64], which is problematic as the animals typically leave the field of view rapidly thereafter. As the green range was used for GFP measurements, we compromised and measured age pigment species that autofluoresce in the red range. Like lipofuscin, we observed these species in gut granules [16] and also in larger gonadal inclusions (Figure S4). Further, we established that our measurements of GFP intensity were not biased by light emitted from the autofluorescent species that also fluoresce in the same wavelengths. We found that the relative degree of autofluorescence was quite low (<10% relative intensity) compared to the GFP signals measured, even in aged animals; correcting for this bleed-through did not alter any findings. Further, the 95^th^-percentile measurements of image intensity we used are less sensitive to low-intensity autofluorescence signals as compared to measures of mean image intensity (for example).

After fluorescence image acquisition, each animal was further stimulated with an 0.25-second pulse of green light, which stimulates a robust escape response in healthy animals and causes head and/or tail retraction in more decrepit individuals. After a 1-second delay, three images were subsequently recorded at 1-second intervals to measure post-stimulation movement.

### Image Analysis

Post-stimulation motion image sequences were visually scrutinized to determine if voluntary “twitching” occurred. Animals with no detectable motion were determined to have died in the interval between the current and previous image acquisition. (Assuming a Bayesian null hypothesis of a uniform prior distribution over time-of-death within the sample interval, the expectation value for the actual, unknown, time of death is halfway between the current and previous acquisition time. We took the number of hours between slide preparation and this time-of-death estimate as the lifespan.) For each timepoint, the non-overexposed fluorescent image with the longest exposure time was selected, subject to manual review to ensure that the animal's locomotion did not cause unacceptable blur. As each fluorescent image was acquired with flanking brightfield images, the brightfield image in which the animal's position is closest to that in the fluorescent image was selected automatically as that with maximum mutual information with the fluorescent image [65], again subject to manual review.

Once the best brightfield/fluorescence image pairs were defined, the outline of the animal in each brightfield image was determined using custom semi-automated software; the position was assumed to be the same in the fluorescent image. The nematode-finding procedure was as follows: based on a training set of labeled nematode/non-nematode regions of brightfield images, a logistic regression classifier was trained to estimate the probability that a given patch of pixels is inside of an animal [66]. The classifier was applied to each brightfield image to create a rough mask, which was distance-transformed to produce “valleys” of low values along the midlines of masked regions. Based on user-input head and tail points, a least-cost path through this distance-transformed mask was calculated using Dijkstra's algorithm. This centerline was manually modified as necessary, and the left and right flanks of each animal determined based on average size for its age, with manual modifications.

Given each animal's centerline and outline, it is trivial to straighten the image and to warp the size and shape of any given animal to a standardized “unit worm” defined by the average size and shape of all animals of a given age. The PCA fluorescence measurements described below were made on standardized, warped images; all other measurements were made from the original images, within the animal's boundaries as defined. Further, given the head-to-tail centerline, we defined the “head” region as the initial 20% of the animal.

### Image Measurements

Fluorescence measurements were made on images corrected for background camera noise (dark field), spatial illumination inhomogeneities (flat field) and temporal variation in illumination via reference fluorescent beads [36]; after correction, intensity values were divided by the exposure time to render all images comparable. Pixel values within the defined whole-animal or head regions were extracted and summary statistics (such as 95^th^ percentile of intensity) made. These raw measurements are available as Table S3, while per-animal summary statistics (days 3–7 slope and mean, etc.) are provided in Table S4.

Four measures of motion were made within the defined animal region: the fraction of pixels changing relative intensity by more than 18% between the different brightfield images acquired, and the average pixel-wise coefficient of variation across these images, both before and after green-light stimulation. ν-support-vector regression (SVR) [67] using an RBF kernel was then used to map these four parameters to the number of days of life remaining, using LIBSVM [68]. Parameters were selected using 10-fold cross-validation on a subset of the input data: C = 10, ν = 0.8, γ = 0.3, though performance was roughly equivalent across several decades of C and γ values, 0.2<ν<0.9. Thereafter we used 100-fold cross-validation on our dataset of 4318 motion-statistics/days-remaining data points and made predictions for each data point without “peeking” by training the SVR on the days-remaining figure for that data point. The predicted “days of life remaining” based on the four measures of motion was used as our aggregate motion score.

For simplicity, image texture features were calculated directly from pixel intensity patterns [69], though “filter-bank” methods have also been employed on nematode images [44], [45]. First, age-specific texture patterns (“textons”) were determined. Brightfield images (acquired through the GFP filterset only) were grouped by age: 3- and 4-day-old, 5- and 6-day-old, up to 15- and 16-day-old. For each group of images, 500000 17×17-pixel patches (within the defined animal outlines) were randomly sampled, after which k-means classification was performed to yield 30 representative textons for each age group (210 overall). Next, the texture of each animal was characterized as follows: for each 17×17-pixel patch falling within the defined brightfield image region, the closest texton (in terms of Euclidian distance) of the 210 overall was determined. The “texture signature” of a given image was defined as the 210-element histogram containing the number of closest-matching 17×17-pixel patches for each texton, divided by the total number of patches in that image. These signatures were then used as input to a support vector regression procedure precisely as described above (best parameters: C = 5, ν = 0.6, γ = 0.004; again performance was relatively insensitive to parameter setting). Texture-based predictions of “days of life remaining” were used as texture-decrepitude scores.

Principal components analysis was performed on fluorescent images, from day 3 onward, warped to unit size and shape. However, as *C. elegans* stretch and compress as they move, and due to inter-individual anatomical variation as well as variation in animal-outline-finding, warping images based on the outline alone does not cause anatomical features to come into precise register across every animal. We therefore manually defined the position of the vulva on each brightfield image and used that position to initialize a mild nonlinear warping procedure, which longitudinally stretches and compresses the image using five evenly spaced control points. Given an image and a reference, hill-climbing optimization was then used to find the position of the control points that maximized the correlation coefficient between the image and reference pixels, with a penalty for large deformations. The *mir-71*::GFP images were mutually aligned using the expectation-maximization algorithm as follows: the mean image across the population was calculated (expectation step), then each image was warped to match the mean (maximization step). These steps were alternated until convergence; typically three iterations sufficed. Results were then manually inspected to ensure face validity. Finally, the mean pixel intensity of each image was subtracted away so that inter-image variability was due only to the distribution of pixel intensities, and not to overall changes in mean brightness. After this procedure, the principal components analysis was performed on the images, and PCA scores along each component were calculated for each animal at each timepoint.

### Statistical Analysis

Estimates of the underlying distribution of sampled data (length, lifespans) were performed with Gaussian kernel density estimation, using the Scott's rule-of-thumb to choose the kernel variance (i.e. bandwidth): σ^2^ *n* ^−0.2^ where σ^2^ is the sample variance and *n* is the sample size [70]. Pairs of lifespan distributions were tested for equality using the two-tailed Kolmogorov–Smirnov test.

Single and multivariate regression of biomarkers versus lifespan was conducted with ordinary least-squares regression, with the coefficient of determination (R^2^) calculated according to the standard formula. Note that in the univariate case, this is equivalent to the squared Pearson product-moment correlation coefficient (*r*) between the biomarker and lifespan. Significance of correlations was measured with an *F*-test of the R^2^ value: the statistic σ^2^_model_/σ^2^_error_ has an F distribution with (*df*_model_, *df*_error_) degrees of freedom, where σ^2^_model_ and σ^2^_error_ are the model and error components of the overall variance, respectively, *df*_model_ = *p*, *df*_error_ = *n-p*-1, *p* is the number of fit parameters, and *n* is the number of observations. As σ^2^_model_ is the sum of squared distances between the predicted values and the mean value, divided by *df*_model_, and σ^2^_error_ is the sum of squared residuals divided by *df*_error_, simple algebra on the definition of the R^2^ value yields F = R^2^ *df*_error_/([1-R^2^] *df*_model_).

Leave-one-out R^2^ values were calculated as follows: given one or more biomarker values for a set of individuals, an ordinary least-squares regression model to predict lifespan from these values was estimated based on the data from each individual save one; then the lifespan of that individual was predicted using that model. This was repeated for each individual. The R^2^ value was calculated from the residuals of the leave-one-out predictions according to the standard formula.

Partial correlation networks (Figure 6B and Figure S6) were computed using TETRAD IV [71], using the “PC” search algorithm with the multiple-regression independence test and the α threshold for the test set to 0.001. Arrows of direction of the influences were discarded, as these inferences were not robust across independence tests or α values; however the basic network structure was robust.

Lifespan-predictive values versus age (Figure 6C) were calculated as follows. For each age *n*, from 2–7 days, all levels measured for a particular marker from day 2 to *n* were considered. (That is, for *n* = 2, only the day-2 value was used; for *n* = 4, the values at days 2, 3, and 4 were used.) The values under consideration, and all of their pairwise multiples (“interaction terms”, so that rates of change can be incorporated) were then used to construct a multivariate regression model to measure how well eventual longevity can be predicted using only data up to age *n*. In order to prevent the changing number of parameters over time from affecting the R^2^ values, and in particular to avoid over-fitting later timepoints due to large number of parameters and interaction terms, we used ridge regression, with a penalty term automatically chosen to minimize the generalized cross-validation error [72].

## Supporting Information

Figure S1 Schematic of image acquisition, processing, and analysis. See Materials and Methods for full details; in brief, image series of brightfield and fluorescence images are acquired for each animal, followed by a bright-light stimulus and three follow-up images to assay response. Animals that do not move post-stimulus are deemed dead. Next, the best fluorescence/brightfield image pairs for each filterset are chosen automatically according to the specified criteria, and the position of the animal is determined in the brightfield image (a procedure known as “image segmentation”) using a custom semi-automated tool. Finally, measurements are made on the images, as described in the text and Materials and Methods.
(PDF)

Figure S2 Comparison of lifespan distributions and survival curves. (A,B) Bivariate regression of each animal's mean length (days 3–7) and the slope of a least-squares fit to the lengths in that time range against eventual longevity yields a “predicted longevity” for each animal that correlates with the actual longevity with an R^2^ of 0.32. Individual animals can be grouped into cohorts with above-average and below-average “predicted longevities” and followed prospectively, as shown by the lifespan distributions (A) and survival curves (B). The lifespan distribution of animals with above-average and below-average predicted longevity based on length slope and mean are significantly different (*p*<10^−19^; Kolmogorov–Smirnov test). Above-average predicted longevity is a 70% sensitive and specific predictor of above-average actual longevity. (C) Lifespan distribution of animals with above-average and below-average predicted longevity based on autofluorescence slope and mean (Figure 2C) differ significantly (*p*<10^−11^). A test for above-average longevity based on whether this prediction is above or below average is 74% sensitive and specific. (D) Lifespan distributions of animals with above-average and below-average predicted longevity, based on slope and mean of the *mir-71*::GFP PC score (Figure 3G), are significantly different (*p*<10^−12^). A test for above-average longevity based on whether this prediction is above or below average is 81% sensitive and specific. (E) Lifespan distributions of animals with high and low *mir-246*::GFP slopes (Figure 5C) are significantly different (*p* = 0.0075). Note that the low- *mir-246*::GFP-slope cohort has a bimodal lifespan distribution: some are nearly as long-lived as the high-slope cohort, while others have a markedly shortened lifespan. A diagnostic test for long lifespan based on these values is 62% sensitive and specific. (F) Lifespan distributions of animals with high and low *mir-239*::GFP slopes (Figure 5F) differ significantly (*p* = 0.016). A diagnostic test is 60% sensitive and specific. (G) Distribution of observed lifespans for animals with above-average and below-average survival indices (Figure 6A) differ significantly (*p*<10^−16^). An above-average survival index is an 79% sensitive and specific indicator of above-average lifespan.
(PDF)

Figure S3 Consistency between trials. (A) Lifespan distributions per trial (see Table S1); distributions each integrate to one so narrower distributions appear higher. (B) Lifepsan distributions per trial, scaled according to the number of animals included in each trial. The overall distribution is shown shaded in grey (for visual reference and on a separate scale). (C) All scatterplots from Figure 1, Figure 2, Figure 3, and Figure 5 are shown with the data points colored according to trial. (The data plotted in Figure 4 was from a single trial.) At right the overall R^2^ and Pearson's correlation coefficient *r* are shown for the (*x, y*) data shown, as well as the *r* values for each individual trial (*r* values are shown so that the direction of the correlation or anti-correlation can be seen directly).
(PDF)

Figure S4 TRITC-channel autofluorescence images. Brightfield and TRITC-channel fluorescence images (see Materials and Methods; excitation = 530–560nm, emission = 590–650nm) of the low- (left) and high-autofluorescence (right) individuals shown in Figure 2A, at 7 days of age. Fluorescent images were corrected according to microscope calibration data (see Materials and Methods) and individually rescaled for easy visualization of relevant structures. The darkest black indicates the same intensity in both images; the brightest white reflects 2.3× higher actual intensities in the right image than the left.
(PDF)

Figure S5 Characterization of *mir-71*::GFP; *daf-16(mu86)*. (A) Lifespan distribution of *mir-71*::GFP; *spe-9(hc88)* reared according to the basic culture protocol (top; data are those shown in Figure 3); *mir-71*::GFP reared according to the FUDR protocol (middle), and *mir-71*::GFP; *daf-16(mu86)* reared according to the FUDR protocol (bottom). The day 3–7 time window for *mir-71*::GFP; *spe-9(hc88)* (shaded) ends after approximately 3% of the animals in that population have died. The shaded windows for the other curves end at approximately the same position on the lifespan distribution; the beginning of the window was adjusted to maintain the same 3:7 (≈0.4:0.6) ratio: this is days 5–13 (middle) and 4–9 (bottom). (B) Population mean ± one standard deviation over time in GFP fluorescence in the head region is shown for *mir-71*::GFP and *mir-71*::GFP; *daf-16(mu86)*. Data were analyzed only between approximately the second and fourteenth day post L1 synchronization. (C) Representative GFP fluorescence images of *mir-71*::GFP and *mir-71*::GFP; *daf-16(mu86)* individuals at day 7. The five individuals with head GFP intensity closest to their population mean were chosen and warped to standard shape and size. Image intensities are shown on the same black-to-white scale for quantitative comparison.
(PDF)

Figure S6 Partial correlation networks for each dataset. Only variables measured for all animals in a given dataset (see Table S1) are shown in each of the above networks, which were generated as specified in the Materials and Methods.
(PDF)

Table S1 Descriptions of each dataset. The number of animals examined and whether texture and age-pigment measurements were made is shown for each individual data-set (which are aggregated in the main text figures and plotted individually in Figure S3).
(PDF)

Table S2 Correlations of biomarkers with lifespan controlling for other markers. The leftmost column shows the correlation R^2^-value of the given measurements versus lifespan, while the other columns show the R^2^ (as a percentage of the original value) after controlling for other variables.
(PDF)

Table S3 Raw timecourse data for datasets.
(CSV)

Table S4 Per-animal summary data for all datasets.
(CSV)
