# Supplementary material for: MicroRNA Predictors of Longevity in Caenorhabditis elegans
Source: PLoS Genet. 2011 Sep 29;7(9):e1002306. doi: 10.1371/journal.pgen.1002306 (PMC3183074; doi:10.1371/journal.pgen.1002306)
Supplement: Figure S1 — Schematic of image acquisition, processing, and analysis. See Materials and Methods for full details; in brief, image series of brightfield and fluorescence images are acquired for each animal, followed by a bright-light stimulus and three follow-up images to assay response. Animals that do not move post-stimulus are deemed dead. Next, the best fluorescence/brightfield image pairs for each filterset are chosen automatically according to the specified criteria, and the position of the animal is determined in the brightfield image (a procedure known as “image segmentation”) using a custom semi-automated tool. Finally, measurements are made on the images, as described in the text and Materials and Methods. (PDF) [file pgen.1002306.s001.pdf]

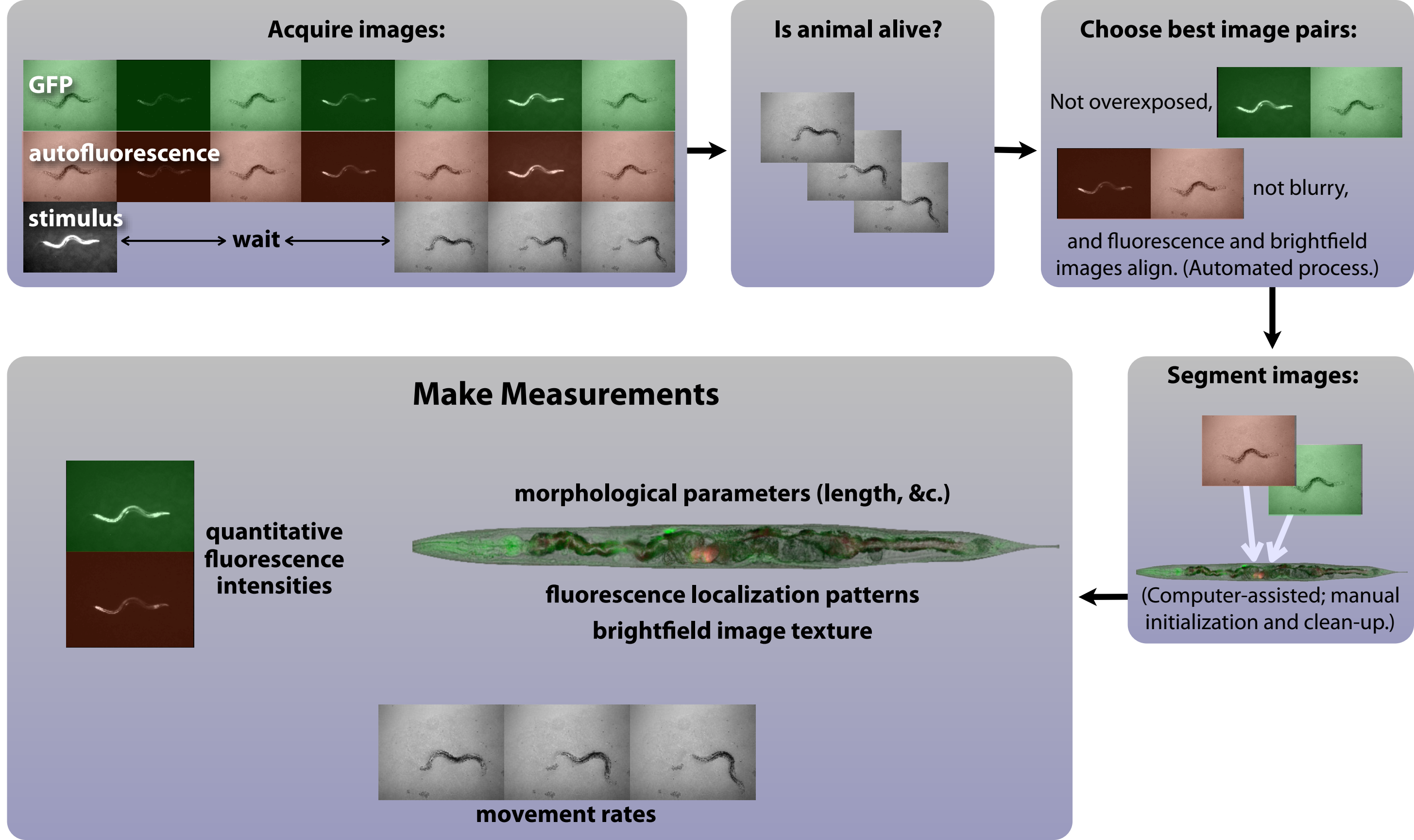

Acquire images:
GFP
autofluorescence
stimulus
wait
Is animal alive?
Choose best image pairs:
Not overexposed,
not blurry,
and fluorescence and brightfield images align. (Automated process.)
Segment images:
(Computer-assisted; manual initialization and clean-up.)
Make Measurements
quantitative fluorescence intensities
morphological parameters (length, &c.)
fluorescence localization patterns
brightfield image texture
movement rates
