# Supplementary figures and images for: MicroRNA Predictors of Longevity in Caenorhabditis elegans
Source: PLoS Genet. 2011 Sep 29;7(9):e1002306. doi: 10.1371/journal.pgen.1002306 (PMC3183074; doi:10.1371/journal.pgen.1002306)

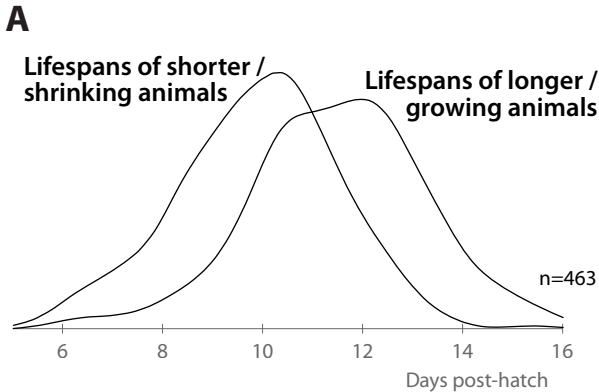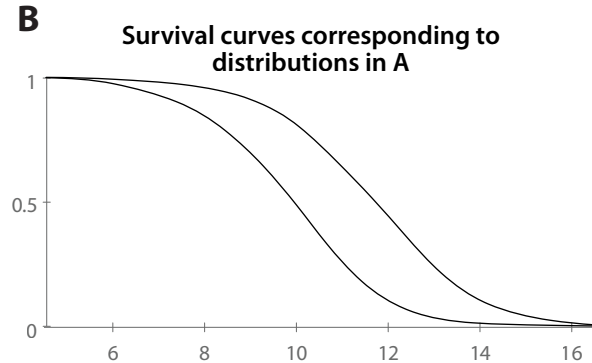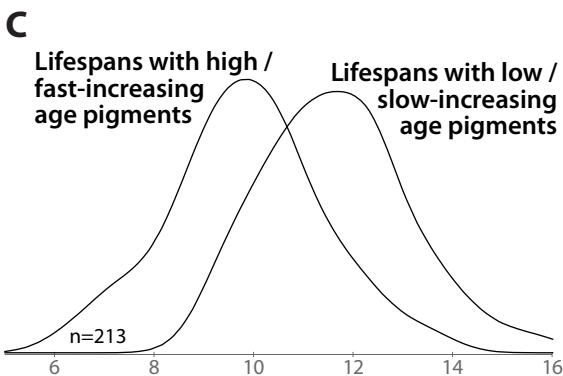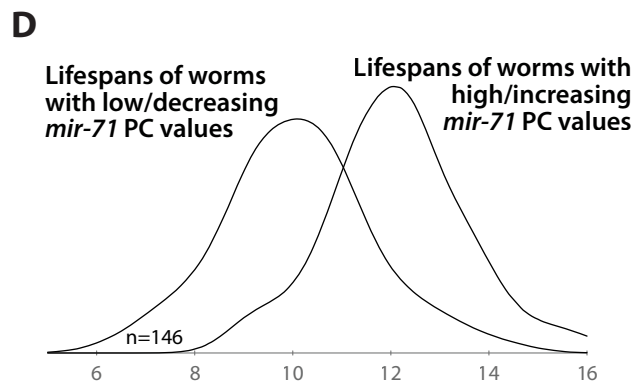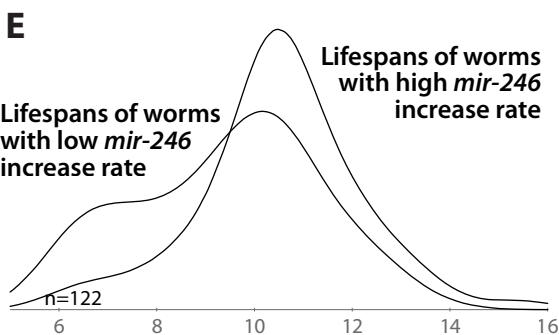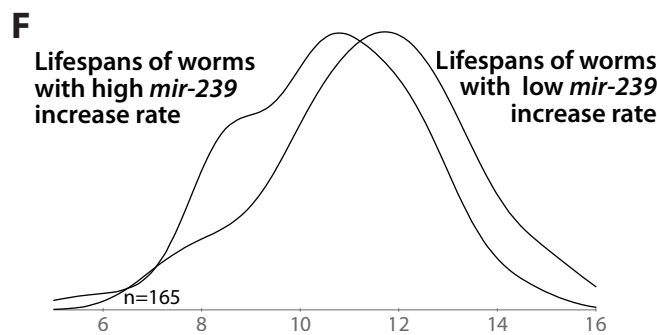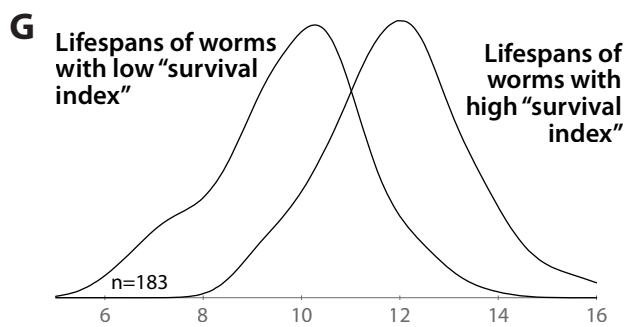

Supplement: Figure S2 — Comparison of lifespan distributions and survival curves. (A,B) Bivariate regression of each animal's mean length (days 3–7) and the slope of a least-squares fit to the lengths in that time range against eventual longevity yields a “predicted longevity” for each animal that correlates with the actual longevity with an R2 of 0.32. Individual animals can be grouped into cohorts with above-average and below-average “predicted longevities” and followed prospectively, as shown by the lifespan distributions (A) and survival curves (B). The lifespan distribution of animals with above-average and below-average predicted longevity based on length slope and mean are significantly different (p<10−19; Kolmogorov–Smirnov test). Above-average predicted longevity is a 70% sensitive and specific predictor of above-average actual longevity. (C) Lifespan distribution of animals with above-average and below-average predicted longevity based on autofluorescence slope and mean (Figure 2C) differ significantly (p<10−11). A test for above-average longevity based on whether this prediction is above or below average is 74% sensitive and specific. (D) Lifespan distributions of animals with above-average and below-average predicted longevity, based on slope and mean of the mir-71::GFP PC score (Figure 3G), are significantly different (p<10−12). A test for above-average longevity based on whether this prediction is above or below average is 81% sensitive and specific. (E) Lifespan distributions of animals with high and low mir-246::GFP slopes (Figure 5C) are significantly different (p = 0.0075). Note that the low- mir-246::GFP-slope cohort has a bimodal lifespan distribution: some are nearly as long-lived as the high-slope cohort, while others have a markedly shortened lifespan. A diagnostic test for long lifespan based on these values is 62% sensitive and specific. (F) Lifespan distributions of animals with high and low mir-239::GFP slopes (Figure 5F) differ significantly (p = 0.016). A diagn [file pgen.1002306.s002.pdf]

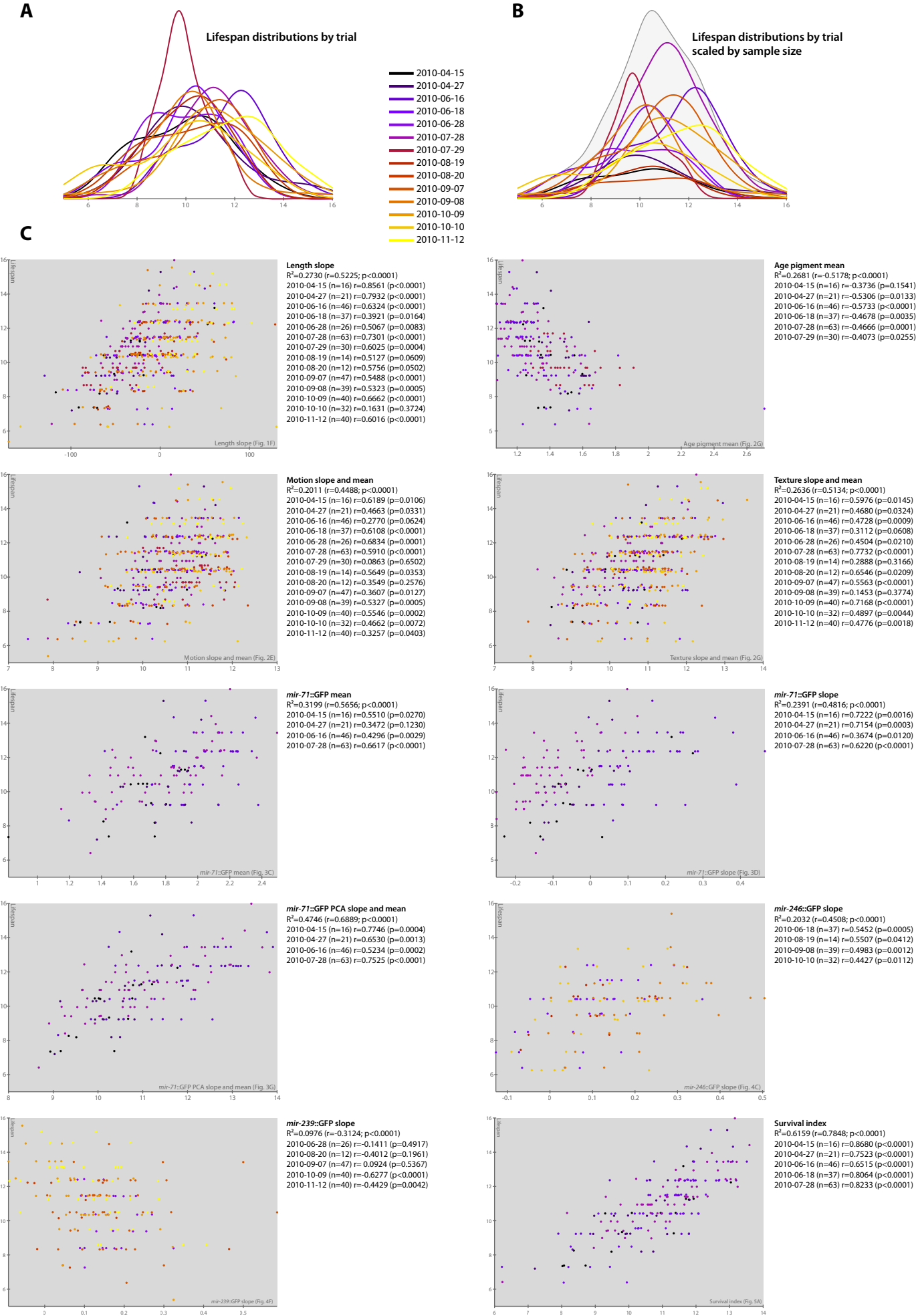

Supplement: Figure S3 — Consistency between trials. (A) Lifespan distributions per trial (see Table S1); distributions each integrate to one so narrower distributions appear higher. (B) Lifepsan distributions per trial, scaled according to the number of animals included in each trial. The overall distribution is shown shaded in grey (for visual reference and on a separate scale). (C) All scatterplots from Figure 1, Figure 2, Figure 3, and Figure 5 are shown with the data points colored according to trial. (The data plotted in Figure 4 was from a single trial.) At right the overall R2 and Pearson's correlation coefficient r are shown for the (x, y) data shown, as well as the r values for each individual trial (r values are shown so that the direction of the correlation or anti-correlation can be seen directly). (PDF) [file pgen.1002306.s003.pdf]

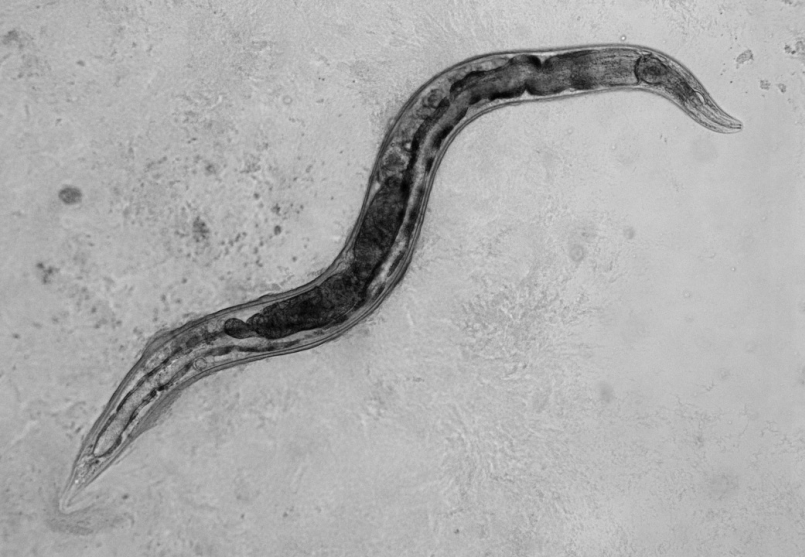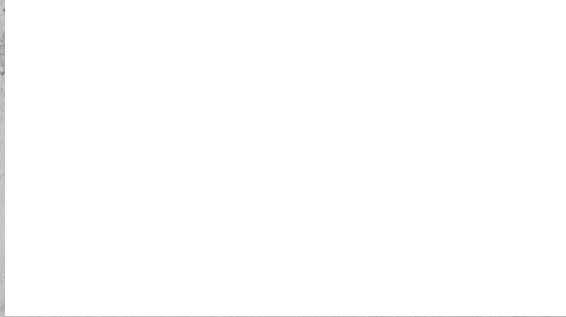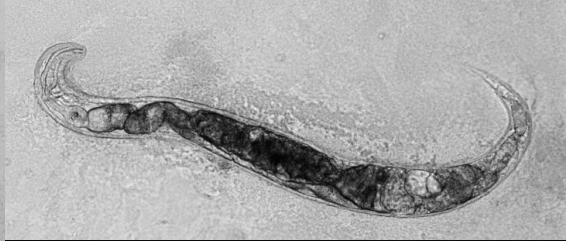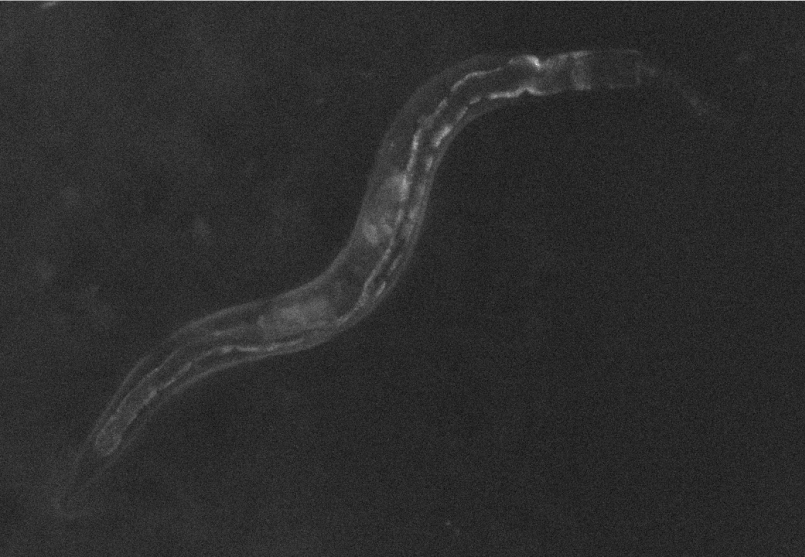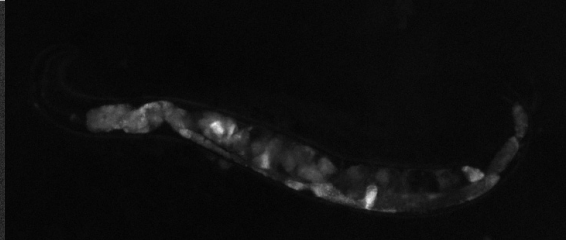

200  $\mu$ m

Supplement: Figure S4 — TRITC-channel autofluorescence images. Brightfield and TRITC-channel fluorescence images (see Materials and Methods; excitation = 530–560nm, emission = 590–650nm) of the low- (left) and high-autofluorescence (right) individuals shown in Figure 2A, at 7 days of age. Fluorescent images were corrected according to microscope calibration data (see Materials and Methods) and individually rescaled for easy visualization of relevant structures. The darkest black indicates the same intensity in both images; the brightest white reflects 2.3× higher actual intensities in the right image than the left. (PDF) [file pgen.1002306.s004.pdf]

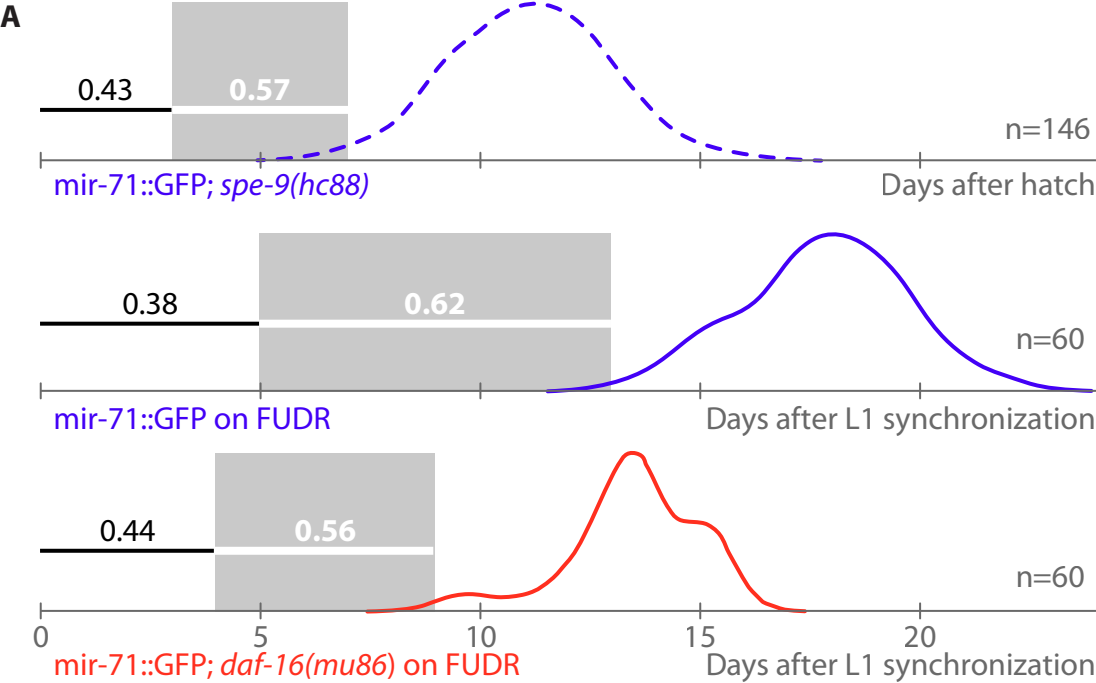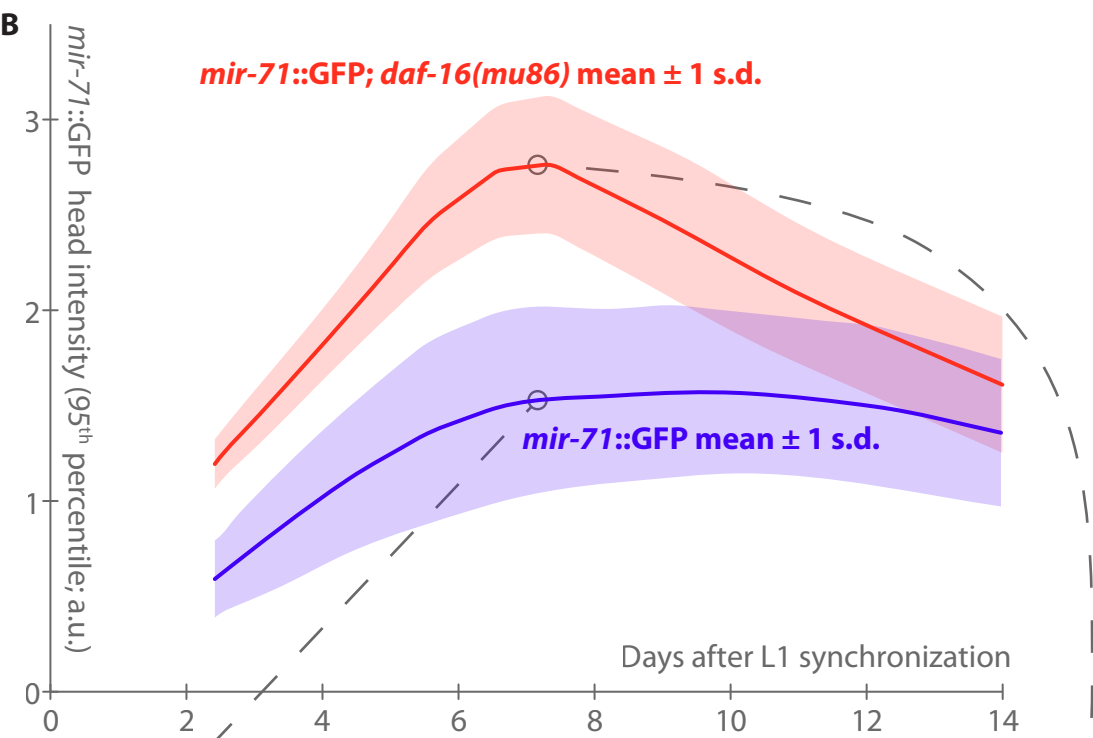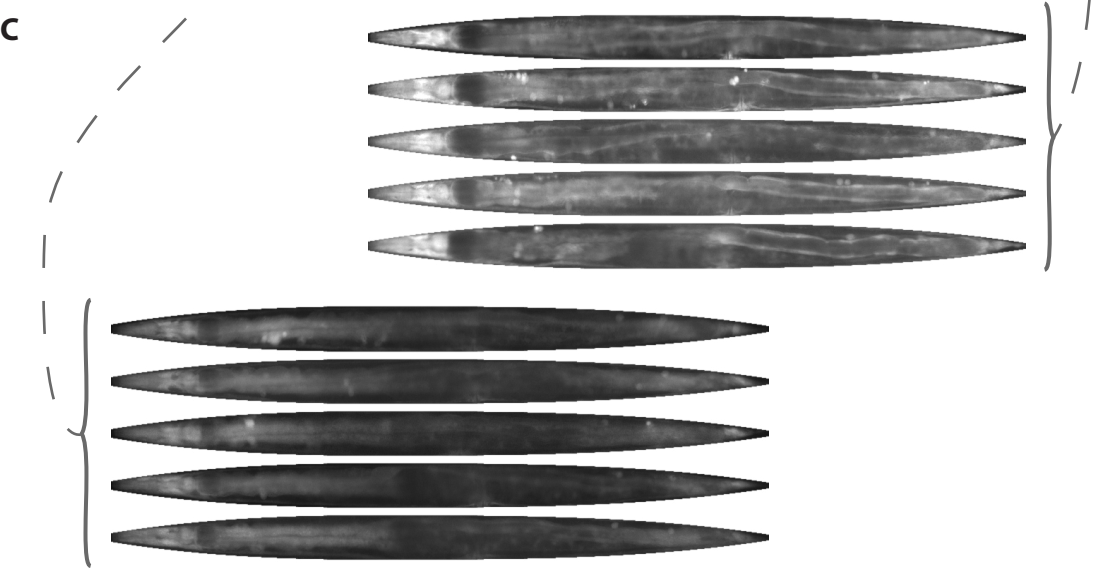

Supplement: Figure S5 — Characterization of mir-71::GFP; daf-16(mu86). (A) Lifespan distribution of mir-71::GFP; spe-9(hc88) reared according to the basic culture protocol (top; data are those shown in Figure 3); mir-71::GFP reared according to the FUDR protocol (middle), and mir-71::GFP; daf-16(mu86) reared according to the FUDR protocol (bottom). The day 3–7 time window for mir-71::GFP; spe-9(hc88) (shaded) ends after approximately 3% of the animals in that population have died. The shaded windows for the other curves end at approximately the same position on the lifespan distribution; the beginning of the window was adjusted to maintain the same 3:7 (≈0.4:0.6) ratio: this is days 5–13 (middle) and 4–9 (bottom). (B) Population mean ± one standard deviation over time in GFP fluorescence in the head region is shown for mir-71::GFP and mir-71::GFP; daf-16(mu86). Data were analyzed only between approximately the second and fourteenth day post L1 synchronization. (C) Representative GFP fluorescence images of mir-71::GFP and mir-71::GFP; daf-16(mu86) individuals at day 7. The five individuals with head GFP intensity closest to their population mean were chosen and warped to standard shape and size. Image intensities are shown on the same black-to-white scale for quantitative comparison. (PDF) [file pgen.1002306.s005.pdf]
