# Supplementary material for: MicroRNA Predictors of Longevity in Caenorhabditis elegans
Source: PLoS Genet. 2011 Sep 29;7(9):e1002306. doi: 10.1371/journal.pgen.1002306 (PMC3183074; doi:10.1371/journal.pgen.1002306)
Supplement: Figure S6 — Partial correlation networks for each dataset. Only variables measured for all animals in a given dataset (see Table S1) are shown in each of the above networks, which were generated as specified in the Materials and Methods. (PDF) [file pgen.1002306.s006.pdf]

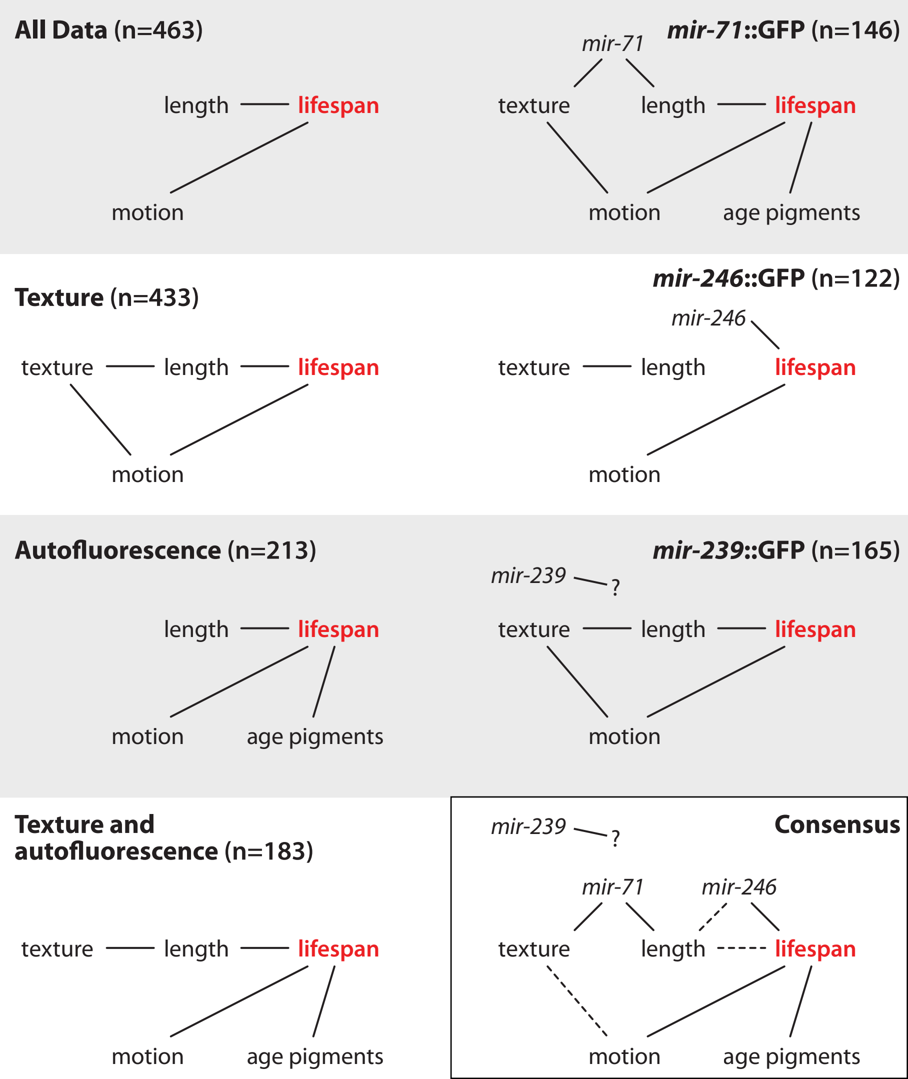

All Data (n=463)
length
lifespan
motion
mir-71::GFP (n=146)
mir-71
texture
length
lifespan
motion
age pigments
Texture (n=433)
texture
length
lifespan
motion
mir-246::GFP (n=122)
mir-246
texture
length
lifespan
motion
Autofluorescence (n=213)
length
lifespan
motion
age pigments
mir-239::GFP (n=165)
mir-239
?
texture
length
lifespan
motion
Texture and autofluorescence (n=183)
texture
length
lifespan
motion
age pigments
Consensus
mir-239
?
mir-71
mir-246
texture
length
lifespan
motion
age pigments
