# Supplementary material for: MicroRNA Predictors of Longevity in Caenorhabditis elegans
Source: PLoS Genet. 2011 Sep 29;7(9):e1002306. doi: 10.1371/journal.pgen.1002306 (PMC3183074; doi:10.1371/journal.pgen.1002306)
Supplement: Table S1 — Descriptions of each dataset. The number of animals examined and whether texture and age-pigment measurements were made is shown for each individual data-set (which are aggregated in the main text figures and plotted individually in Figure S3). (PDF) [file pgen.1002306.s007.pdf]

Date	Animals	Transgene	Texture?	Age Pigments?
2010-04-15	16	*mir-71*::GFP	Yes	Yes
2010-04-27	21	*mir-71*::GFP	Yes	Yes
2010-06-16	46	*mir-71*::GFP	Yes	Yes
2010-06-18	37	*mir-246*::GFP	Yes	Yes
2010-06-28	26	*mir-239*::GFP	Yes	No
2010-07-28	63	*mir-71*::GFP	Yes	Yes
2010-07-29	30	*mIs10*	No	Yes
2010-08-19	14	*mir-246*::GFP	Yes	No
2010-08-20	12	*mir-239*::GFP	Yes	No
2010-09-07	47	*mir-239*::GFP	Yes	No
2010-09-08	39	*mir-246*::GFP	Yes	No
2010-10-09	40	*mir-239*::GFP	Yes	No
2010-10-10	32	*mir-246*::GFP	Yes	No
2010-11-12	40	*mir-239*::GFP	Yes	No

Texture was not calculated for *mIs10* animals: for these, extremely bright head GFP was captured in the brightfield images (which were acquired with the fluorescent shutter open for technical reasons). Other transgenes did not express GFP at levels high enough to alter the brightfield textural appearance.
