# Supplementary material for: MicroRNA Predictors of Longevity in Caenorhabditis elegans
Source: PLoS Genet. 2011 Sep 29;7(9):e1002306. doi: 10.1371/journal.pgen.1002306 (PMC3183074; doi:10.1371/journal.pgen.1002306)
Supplement: Table S2 — Correlations of biomarkers with lifespan controlling for other markers. The leftmost column shows the correlation R2-value of the given measurements versus lifespan, while the other columns show the R2 (as a percentage of the original value) after controlling for other variables. (PDF) [file pgen.1002306.s008.pdf]

	Overall R^2 vs. lifespan	**Controlling for the below feature reduces the R^2 vs. lifespan to the given percentage of the overall R^2** (*p* and n values reported are for the controlled correlation)						
		Length	Motion	Texture	Age pigments	*mir-71*::GFP PCA	*mir-246*::GFP	*mir-239*::GFP
**Length**	0.321 (p<0.0001 n=463)		73.5% (p<0.0001 n=463)	35.1% (p<0.0001 n=433)	99.4% (p<0.0001 n=213)	59.1% (p<0.0001 n=146)	15.9% (p=0.0124 n=122)	120.0% (p<0.0001 n=165)
**Motion**	0.201 (p<0.0001 n=463)	50.4% (p<0.0001 n=463)		48.0% (p<0.0001 n=433)	62.7% (p<0.0001 n=213)	24.9% (p=0.0066 n=146)	95.0% (p<0.0001 n=122)	54.1% (p<0.0001 n=165)
**Texture**	0.264 (p<0.0001 n=433)	18.9% (p<0.0001 n=433)	54.8% (p<0.0001 n=433)		101.4% (p<0.0001 n=183)	10.7% (p=0.0427 n=146)	22.4% (p=0.0070 n=122)	97.1% (p<0.0001 n=165)
**Age pigments**	0.311 (p<0.0001 n=213)	59.7% (p<0.0001 n=213)	73.8% (p<0.0001 n=213)	66.9% (p<0.0001 n=183)		35.7% (p<0.0001 n=146)	165.1% (p<0.0001 n=37)*	
***mir-71*::GFP PCA**	0.475 (p<0.0001 n=146)	4.8% (p=0.0684 n=146)	67.2% (p<0.0001 n=146)	30.0% (p<0.0001 n=146)	70.9% (p<0.0001 n=146)			
***mir-246*::GFP**	0.203 (p<0.0001 n=122)	65.5% (p<0.0001 n=122)	63.7% (p<0.0001 n=122)	97.4% (p<0.0001 n=122)	235.5% (p<0.0001 n=37)*			
***mir-239*::GFP**	0.098 (p<0.0001 n=165)	12.1% (p=0.1643 n=165)	54.0% (p=0.0030 n=165)	32.1% (p=0.0230 n=165)				

All correlations include days 3–7 mean and slope values, except for *mir-246* and *mir-239*, which only use the slope measure.

* If *a* and *b* independently determine *c*, controlling for *b* will increase the explanatory power of *a* vs. *c*, and vice-versa. (Other more complex scenarios are also possible.) This may therefore explain the >100% values observed, which is consistent with the networks illustrated in Figs. 6b and S6. The dataset with *mir-246*::GFP and age pigment values was too small to reliably calculate an overall partial-correlation network, so age pigment slope is not directly placed in the *mir-246*::GFP network in Fig. S6; however the >100% controlled-correlation values suggest that *mir-246* influences longevity completely independently from autofluorescence accumulation.
